# Comparative phylogeography of eight herbs and lianas (Marantaceae) in central African rainforests

**DOI:** 10.3389/fgene.2014.00403

**Published:** 2014-11-19

**Authors:** Alexandra C. Ley, Gilles Dauby, Julia Köhler, Catherina Wypior, Martin Röser, Olivier J. Hardy

**Affiliations:** ^1^Institut für Geobotanik und Botanischer Garten, University Halle-WittenbergHalle (Saale), Germany; ^2^Evolutionary Biology and Ecology, Faculté des Sciences, Université Libre de BruxellesBrussels, Belgium

**Keywords:** endemism, distinctiveness, genetic diversity, *trnC-petN1r*, refugia, Lower Guinea

## Abstract

Vegetation history in tropical Africa is still to date hardly known and the drivers of population differentiation and speciation processes are little documented. It has often been postulated that population fragmentations following climate changes have played a key role in shaping the geographic distribution patterns of genetic diversity and in driving speciation. Here we analyzed phylogeographic patterns (chloroplast-DNA sequences) within and between eight (sister) species of widespread rainforest herbs and lianas from four genera of Marantaceae (*Halopegia*, *Haumania, Marantochloa, Megaphrynium*), searching for concordant patterns across species and concordance with the Pleistocene refuge hypothesis. Using 1146 plastid DNA sequences sampled across African tropical lowland rainforest, particularly in the Lower Guinean (LG) phytogeographic region, we analyzed intra- and interspecific patterns of genetic diversity, endemism and distinctiveness. Intraspecific patterns of haplotype diversity were concordant among most species as well as with the species-level diversity pattern of Marantaceae. Highest values were found in the hilly areas of Cameroon and Gabon. However, the spatial distribution of endemic haplotypes, an indicator for refuge areas in general, was not congruent across species. Each proposed refuge exhibited high values of endemism for one or a few species indicating their potential role as area of retraction for the respective species only. Thus, evolutionary histories seem to be diverse across species. In fact, areas of high diversity might have been both refuge and/or crossing zone of recolonization routes i.e., secondary contact zone. We hypothesize that retraction of species into one or the other refuge happened by chance depending on the species' distribution range at the time of climate deterioration. The idiosyncratic patterns found in Marantaceae species are similar to those found among tropical tree species, especially in southern LG.

## Introduction

Vegetation history in tropical Africa is still to date hardly known and the drivers of speciation and population differentiation processes are little documented. Hypotheses on the diversification of the Afrotropical flora include allopatric differentiation/speciation driven by population fragmentation following Pleistocene climate changes (Robbrecht, 1994; Sosef, 1994; Maley, 1996) and parapatric differentiation/speciation across ecological gradients (e.g., temperature and precipitation gradients; Fjeldsa and Lovett, 1997; Vande Weghe, 2004; Heuertz et al., 2013). Phylogeographic studies within and between closely related species might shed new light on this matter as indicated by similar studies in temperate regions (Taberlet et al., 1998; Schönswetter et al., 2005).

Palynological studies (Maley and Brenac, 1998; Dupont et al., 2000; Bonnefille, 2007; Ngomanda et al., 2009; Dupont, 2011) and palaeo-environmental reconstructions (Anhuf et al., 2006) suggest a repeated fragmentation of the tropical forest in Africa due to (glacial-interglacial) climate oscillations for the last million years. For example, during the African Humid Holocene period (c. 6000–9000 years BP) a single forest block extended from West to Central Africa beyond the current forest cover limit, while the forest was presumably highly fragmented and reduced in size during the last glacial maximum (c. 19000–26000 years BP). This might have led to population fragmentation followed by the independent evolution of the isolated populations through mutation and drift and ultimately the establishment of species. Alternatively, and/or simultaneously, isolated populations might have adapted to different climatic conditions ultimately forming ecologically different species. Indeed, within Lower Guinea (LG, i.e., the western part of the Central African rainforest block, identified as a phytochorion by White, 1979, Figure 1), climatic heterogeneity is characterized by a marked W-E precipitation gradient from the Coast to the inland and a North-South seasonal inversion at a latitude c. 2°N (Leroux, 1983; Vande Weghe, 2004).

**Figure 1:**
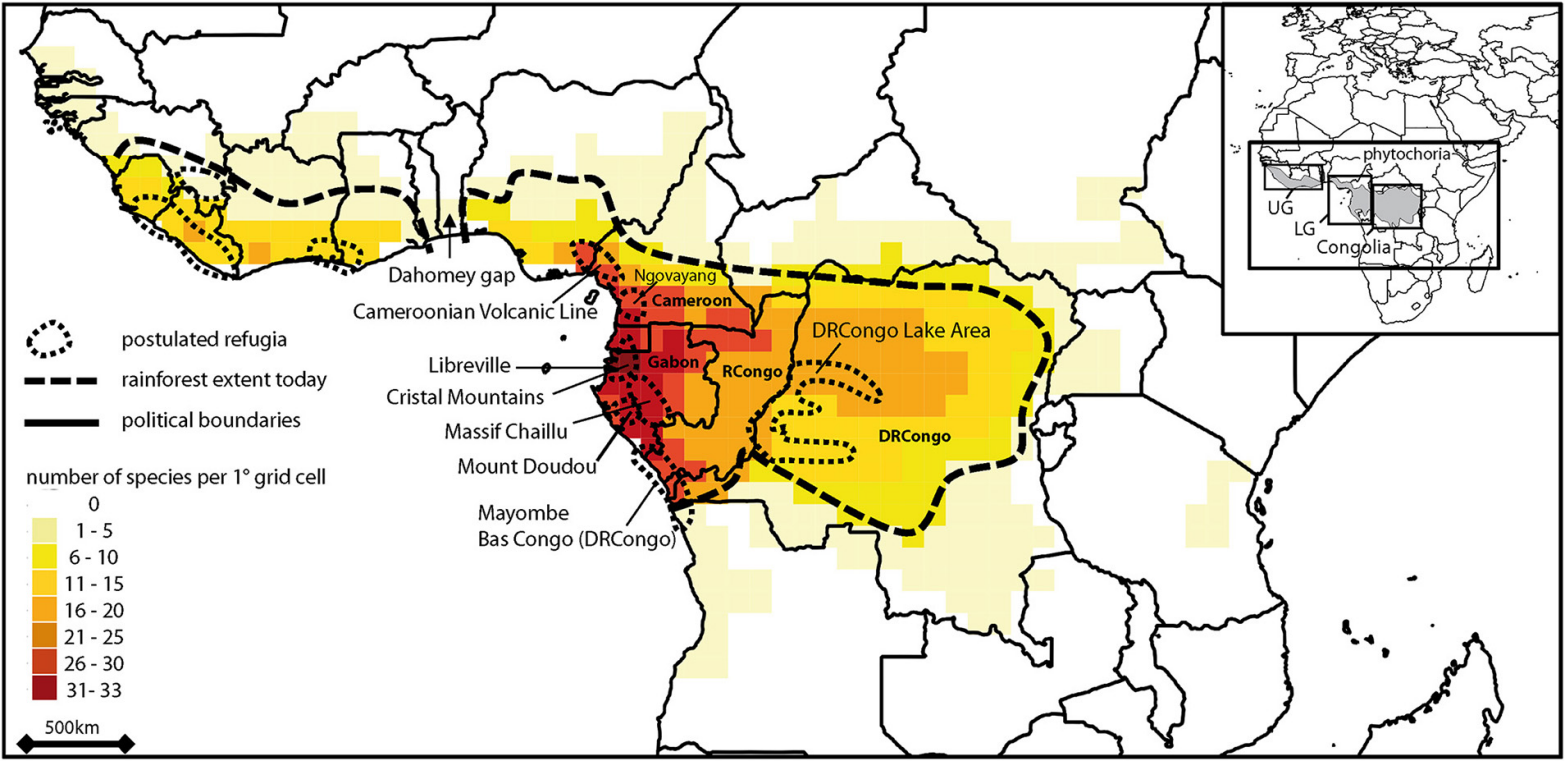
**Pattern of species diversity of Marantaceae in tropical Africa based on Schnell (1957), Dhetchuvi (1996), Jongkind (2008) and Ley and Claßen-Bockhoff (2012)**. Postulated refugia after (Maley, 1996); DRCongo, Democratic Republic of Congo; RCongo, Republic of the Congo. Inset shows phytochoria after (White, 1979): UG, Upper Guinea; LG, Lower Guinea; C, Congolia.

Recently, comparative phylogeographic studies of central African trees revealed a partial congruence of phylogeographic patterns with postulated refugia (Hardy et al., 2013; Heuertz et al., 2013; Dauby et al., 2014a). However, despite the occurrence of some common phylogeographic features, each species displayed an original pattern, especially in Gabon, suggesting idiosyncratic evolutionary histories. It has been hypothesized that this is due to less severe changes in forest cover reduction in this area during climate oscillations (Dupont et al., 2000; see also Holstein and Renner, 2011).

Here we investigate whether similar genetic patterns as so far detected in tropical African trees might also be found in perennial herbs and lianas from the forest understorey. We might expect that, compared to trees, phylogeographic patterns of herbs and lianas mirror younger historical events due to presumably shorter life cycles (Putz, 1990; Gerwing, 2004; Brandes et al., 2011). Furthermore, general patterns might be more structured in herbs than in trees (see e.g., *G*_ST_ in nuclear markers in Nybom, 2004) due to a more patchy community structure and potentially smaller dispersal distances of pollinators and dispersers in the tropical understorey (for trees: <14 km, Ward et al., 2005; 100 m–100 km, Carbone et al., 1999; for understorey shrub: 10–20 m, Zeng et al., 2012).

More specifically, we perform in Lower Guinea a comparative phylogeographic study of eight perennial herbs and lianas of the family Marantaceae. We search for (1) congruent patterns across species that might have been driven by a common vegetation history, and (2) congruence of these patterns with postulated rainforest refugia that might support the importance of these areas for species survival and population differentiation.

## Materials and methods

### Species studied and sampling

The Marantaceae (30 genera) are a pantropical family of perennial herbs and lianas of the understorey and gaps of lowland rainforest (0–1500 m) with highest species diversity found in America (~450 spp.) followed by Asia (~50 spp.) and Africa (~40 spp.) (Dhetchuvi, 1996; Andersson, 1998; Kennedy, 2000; Suksathan et al., 2009; Ley and Claßen-Bockhoff, 2011). Each genus of the family is endemic to one continent except *Halopegia* and *Thalia* (Andersson, 1998). Phylogenetic investigations suggest a split of this family from its sister family Cannaceae some 95 ± 5 Ma ago. The family then started to diversify ca. 63 ± 5 Ma ago (Kress and Specht, 2005) in the late cretaceous with the establishment of the first tropical everwet habitats in the current tropics (Willis and McElwain, 2002). The Marantaceae are thus probably not a Gondwanan group, i.e., Marantaceae are not distributed pantropically due to vicariant events ca. 110 Ma ago (Kearey and Vine, 1996). Biogeographic analyses suggest instead the occurrence of several independent dispersal events between continents followed each time by intra-continental speciation resulting in several independent species clades per continent (Prince and Kress, 2006). In continental Africa the current distribution of the Marantaceae family ranges from Senegal in the West to Tanzania in the East following today's limits of the tropical rainforest. Highest species numbers are found in Gabon and Cameroon (Figure 1). Distribution ranges of individual species vary from widespread (equaling the distribution of the whole Marantaceae family in Africa) to restricted, either to the West or East of the Dahomey gap and/or to Cameroon and/or Gabon (Dhetchuvi, 1996).

The Marantaceae species differ from their sister family Cannaceae by a pulvinus and an explosive pollination mechanism (Claßen-Bockhoff, 1991; Kennedy, 2000). It is a highly diverse family with regard to species number and adaptations to different pollinators and dispersal agents (Kennedy, 2000; Clausager and Borchsenius, 2003; Locatelli et al., 2004; Ley, 2008; Ley and Claßen-Bockhoff, 2009). The species of Marantaceae show typical characteristics of plants from the tropical understory such as self-compatibility (*Halopegia azurea* even autogamous, Ley and Claßen-Bockhoff, 2013), clonality via rhizomes (*Marantochloa congensis* additionally via vivipary (bulbils), Kennedy, 2000) and animal pollination and dispersal (Ley, 2008; Ley and Claßen-Bockhoff, 2009).

For the current study eight species from four different genera with different growth forms, distribution ranges and pollinators were chosen (Table 1). Sampling of leaf material for genetic analyses was envisioned to cover the whole distribution area of each species. However, for all species sampling was better in Cameroon and Gabon and only fragmentary in West Africa (i.e., Upper Guinean phytochorion) and the Congo Basin (i.e., Congolian phytochorion). We thus here used the entire dataset including West Africa and the Congo Basin for the description of the phylogeographic pattern of each species and then limited the dataset to Lower Guinea, when comparing the phylogeographic pattern qualitatively and quantitatively among species.

**Table 1:** **Ecological information on the eight study species from the family Marantaceae**.

**Genera**	**Species**	**Abbr**.	***N* of species studied (total per genus°^+^^#^)**	**Distribution range^+^**	**Growth form^+^**	Pollinator^*^	**Dispersal agent^1^°**
*Halopegia*	*azurea*	HaloAzu	1(1)	GC	herb	Bee (Halictidae)	no data
*Haumania*	*danckelmaniana liebrechtsiana*	HauDanck HauLieb	2(3)	C, LG	liana	Bee (*Xylocopa*)	gravity
*Marantochloa*	*congensis incertifolia monophylla*	MarCong MarIncert MarMono	3(16)	GC, LG, C, LG	herb	Bee (*Amegilla*)	water, monkeys
*Megaphrynium*	*macrostachyum trichogynum*	MegaMacro MegaTrich	2(4)	GC, LG	herb	Bee + Bird	monkeys

Abbr., Abbreviation; N, number;

# Schnell, 1957;

1 Tutin and Fernandez, 1993;

+ Dhetchuvi, 1996;

° Ley, 2008;

* Ley and Claßen-Bockhoff, 2009.

*Phytochoria after White, 1979: C, Congolia; GC, Guineo-Congolian; LG, Lower Guinea; UG, Upper Guinea*.

### DNA extraction and amplification

For the eight species we used sequences from the chloroplast (cp) inter-genic spacer *trnC-petN1r* using the primers trnC 5′-CCAGTTCAAATCTGGGTGTC-3′ (modified from Demesure et al., 1995) and petN1r 5′-CCCAAGCAAGACTTACTATATCC-3′ (Lee and Wen, 2004). For *Marantochloa congensis* an additional marker (*psbA-trnH*) was amplified to increase resolution, using the primers psbA 5′-GTTATGCATGAACGTAATGCTC-3′ and trnH2 5′-CGCGCATGGTGGATTCACAATCC-3′ (Sang et al., 1997; Tate and Simpson, 2003). The genetic data for the genera *Haumania* and *Marantochloa* was updated from Ley and Hardy (2010, 2014). For the third species of the genus *Haumania* (*H. leonardiana*) only sequences from six individuals from the Democratic Republic of Congo (DRCongo) could so far be obtained and were added to the haplotype network to show intragenus relationships but were not analyzed any further due to the scarcity of available sequences. The phylogeographic patterns of three species from the genera *Halopegia* and *Megaphrynium* were characterized here for the first time. The production of sequences for these species followed the protocol of DNA extraction, amplification and sequencing described in Ley and Hardy (2010).

### Geographic distribution of chloroplast haplotypes and phylogenetic networks

For each species chloroplast haplotypes were analyzed in DnaSP Version 5.10 (Librado and Rozas, 2009) and their geographic distribution mapped. DNA haplotypes were submitted to Genbank (for accession numbers see Supplementary Table 1). To obtain the minimum number of mutations between haplotypes, a network was established with the software Network 4.5.1.0 (www.fluxus-engineering.com; Bandelt et al., 1999) using a maximum parsimony method based on a median joining algorithm (MJ). Networks were established per species and for entire genera to identify possible plastid captures between closely related species (Ley and Hardy, 2010, 2014). Nucleotide diversity, which represents the average number of nucleotide differences per site between two sequences, was calculated in Arlequin (Excoffier et al., 2009).

### Grid-based standardized measures of genetic diversity, endemism and distinctiveness

For the comparison of geographic patterns of genetic diversity between species at different scales in Lower Guinea we subdivided the region into three different grid systems with cell sizes of 0.75°-, 1.5°- and 3°-sides (Supplementary Figure 1 for 0.75° and 1.5°; 3° not shown). Given that a minimum of three samples was necessary per species and grid cell to compute diversity indices (see below), smaller cells allowed higher spatial resolution but at the cost of lower precision and higher loss of data in areas were sampling was less dense (for numbers of individuals per grid cell 0.75° and 1.5° see Supplementary Tables 2, 3).

#### Within cell diversity, endemism and distinctiveness

We computed several statistics quantifying genetic diversity for each species within each cell: Nielsen's estimator of the effective number of haplotypes *NAe* (Nielsen et al., 2003), the gene diversity corrected for sample size *He* (Nei, 1978) and the mean phylogenetic distance between individuals *v* (gene diversity with ordered alleles, Pons and Petit, 1996). The different statistics were computed with SPAGeDi Version 1.4 (Hardy and Vekemans, 2002). The degree of endemism of each haplotype was assessed by the maximal distance between individuals carrying that haplotype. We quantified the degree of haplotypic endemism, *End*, per cell and species as the proportion of individuals carrying haplotypes with a maximal geographic extension of 200 km. Finally, for each species, the level of phylogenetic distinctiveness of each cell with respect to the other ones was computed following Dauby et al. (2014a). To this end, for each pair of cells (*i* and *j*), the mean phylogenetic distance between individuals drawn from *i* and *j* (*v_ij_*) was computed, as well as the spatial distance between the centroids of individuals belonging to *i* and *j* (*d_ij_*). *S'_ij_*, the residuals of the regression of *v_ij_* on ln(*d_ij_*), or the centered *v_ij_* values themselves if there was no significant positive correlation between *v_ij_* and ln(*d_ij_*) according to a Mantel test, were then averaged over all pairs involving one particular cell, *S'_i_*, providing a measure of the phylogenetic distinctiveness of that cell above or below the average across all cells (Petit et al., 2003; Dauby et al., 2014a).

#### Differentiation statistics

Global differentiation statistics *G*_ST_ and *N*_ST_ among cells (for cells with at least three individuals) were computed for each species. *G*_ST_ accounts for differences in haplotype frequencies while *N*_ST_ additionally accounts for the phylogenetic distances between haplotypes. To test if there was a phylogeographic signal, characterized by *N*_ST_ > *G*_ST_, permutation tests were performed in SPAGeDi Version 1.4 (Hardy and Vekemans, 2002).

### Congruence of phylogeographic patterns among species

Congruence of phylogeographic patterns for each pair of species was evaluated (i) by comparing within cell diversity and endemism metrics using Pearson correlation tests, and (ii) by comparing matrices of pairwise standardized distinctiveness among grid cells (*S'_ij_*) using Mantel tests (see Dauby et al., 2014a). To obtain a multispecies test of overall geographic congruence of local diversity, endemism or distinctiveness, these metrics were first centered (i.e., minus their mean value) and reduced (i.e., divided by their standard deviation) within species, and then differences among grid cells were tested using a One-Way ANOVA where grid cells were used as factor (due to missing data, species could not be added as another factor). To represent diversity patterns on a map, centered and reduced diversity and endemism metrics were shown per cell and species, or were averaged over species to represent multi-species trends.

## Results

### Genetic polymorphism

The numbers of individuals sequenced per species ranged from 75 to 166, totaling 1046 individuals (991 in Lower Guinea, Table 2) sequenced for *trnC-petN1r* and 110 for *psbA-trnH* (Lower Guinea only, Table 2).

**Table 2:** **Sample sizes and genetic diversity at the *trnC-petN1r* and *psbA-trnH2* region in the eight study species from the Marantaceae**.

**Species**	***N* (whole tropical Africa/Lower Guinea)**	**SNP (incl.indels)**	**Nucleotide diversity × 10^3^**	***N* of haplotypes**	
				**whole tropical Africa (total/private)**	**Lower Guinea (total/private)**
***trnC-petN1r***					
HaloAzu	121/115	11	0.846 ± 0.690	11	8
HauDanck	121/121	16	1.575 ± 1.060	14/10	14/10
HauLieb	110/110	20	3.268 ± 1.894	10/6	10/6
MarCong	194/169	61	3.619 ± 2.149	15/11	15/9
MarIncert	81/81	22	6.901 ± 3.755	13/6	13/6
MarMono	118/109	60	7.289 ± 3.925	19/16	19/15
MegaMacro	162/162	22	4.897 ± 2.852	16/15	16/15
MegaTrich	131/131	13	1.022 ± 0.900	7/6	7/6
Total	1038/998				
***psbA-trnH2***	110/110	9	5.754 ± 3.152	7	7
MarCong					

For abbreviations of species names see Table 1. N, number of individuals; SNP, number of single nucleotide polymorphisms. Haplotypes (total/private): “total” includes haplotypes shared between sister species from the same genus. “Private” considers only haplotypes found in the respective species.

The *trnC-petN1r* region had an average length of about 800 bp (including indels). The number of SNPs (counting indels as single mutations) per species varied between 11 (*Halopegia azurea*) and 27 (*Marantochloa monophylla*) and the number of haplotypes per species varied between seven (*Megaphrynium trichogynum*) and 19 (*Marantochloa monophylla*, see Table 2). Overall nucleotide diversity ranged from 0.000846 ± 0.000690 in *Halopegia azurea* to 0.007289 ± 0.003925 in *Marantochloa monophylla* (Table 2). Average genetic diversity per grid cell measured as *He* was highest in *Haumania danckelmaniana* (Table 3). Genetic diversity measured as *v* taking genetic distance between haplotypes into account was highest in *Marantochloa monophylla* and endemism per grid cell (*End*) was highest in *M. incertifolia*. Measures of genetic diversity were independent of grid cell size (Supplementary Table 4).

**Table 3:** **Within cell diversity pattern at the *trnC-petN1r* region in eight Marantaceae species in Lower Guinea for grid cell size 0.75° (average across grid cells, [range])**.

**Species**	**Total sample size**	***N* cells with >2 individuals**	**Mean [range] per cell of**				
			***N* individuals**	***NAe***	***He***	***v***	***End***
HalAzu	91	18	5.06 [3,11]	1.41 [1,3.13]	0.19 [0,0.71]	0.26 [0,0.86]	0.09 [0,1]
HauDanck	115	19	6.05 [3,15]	1.92 [1,10]	0.37 [0,1]	0.63 [0,2.2]	0.16 [0,0.8]
HauLieb	98	15	6.53 [3,16]	1.36 [1,3.04]	0.21 [0,0.67]	0.81 [0,5]	0.11 [0,0.54]
MarCong	141	21	6.71 [3,15]	1.61 [1,5.05]	0.30 [0,1]	0.84 [0,7.33]	0.10 [0,1]
MarIncert	73	9	8.11 [3,20]	1.61 [1,3.57]	0.24 [0,0.8]	1.04 [0,3.69]	0.55 [0,1]
MarMono	95	18	5.28 [3,12]	1.76 [1,4.61]	0.27 [0,1]	1.29 [0,8]	0.12 [0,0.78]
MegaMacro	140	22	6.36 [3,22]	1.72 [1,4.61]	0.34 [0,1]	0.99 [0,4]	0.17 [0,1]
MegaTrich	113	21	5.38 [3,11]	1.50 [1,2.27]	0.29 [0,0.67]	0.38 [0,2.16]	0.02 [0,0.2]

N, number; NAe, Effective N of alleles (Nielsen et al., 2003); He, gene diversity corrected for sample size (Nei, 1978); v, mean phylogenetic distance between individuals (Pons and Petit, 1996); End, mean proportion of individuals carrying endemic alleles (haplotype range < 200 km). For abbreviations of species names see Table 1.

For *M. congensis psbA-trnH* sequences reached a length of about 900 bp (including indels and reverse mutations). Reverse mutations were excluded in the following analyses leaving nine mutations, seven haplotypes (Table 2) and a network without loops with a maximum of two mutations between adjacent haplotypes (Supplementary Figure 2).

**Figure 2:**
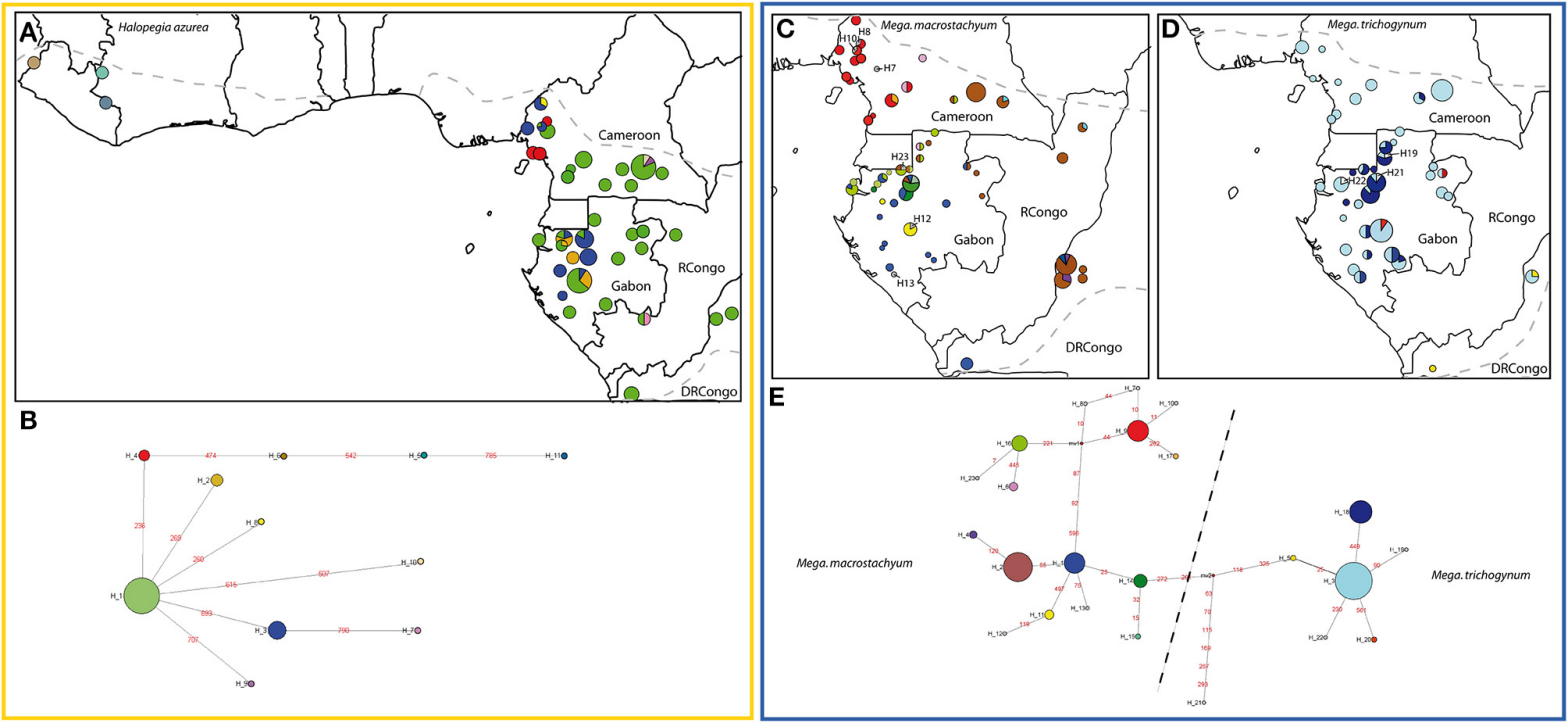
**Geographic distribution of chloroplast haplotypes and generic haplotype networks based on *trnC-petN1r* for the following Marantaceae species**. *Halopegia azurea* **(A,B)**, *Megaphrynium macrostachyum* **(C,E)**, *Mega. trichogynum*, **(D,E)**. Gray hatched line: species distribution range. Sizes of circles are proportional to sample sizes at each locality in the geographic maps and proportional to haplotype frequency in the haplotype network. Frequent haplotypes are color-coded, rare haplotypes are number-coded. Stippled lines throughout the networks delineate groups of haplotypes according to the species in which they are usually found, but haplotypes can also be shared among species. Red numbers along branches are IDs of mutations, mv1 to mv2 indicate median vectors (Bandelt et al., 1999).

**Figure 3:**
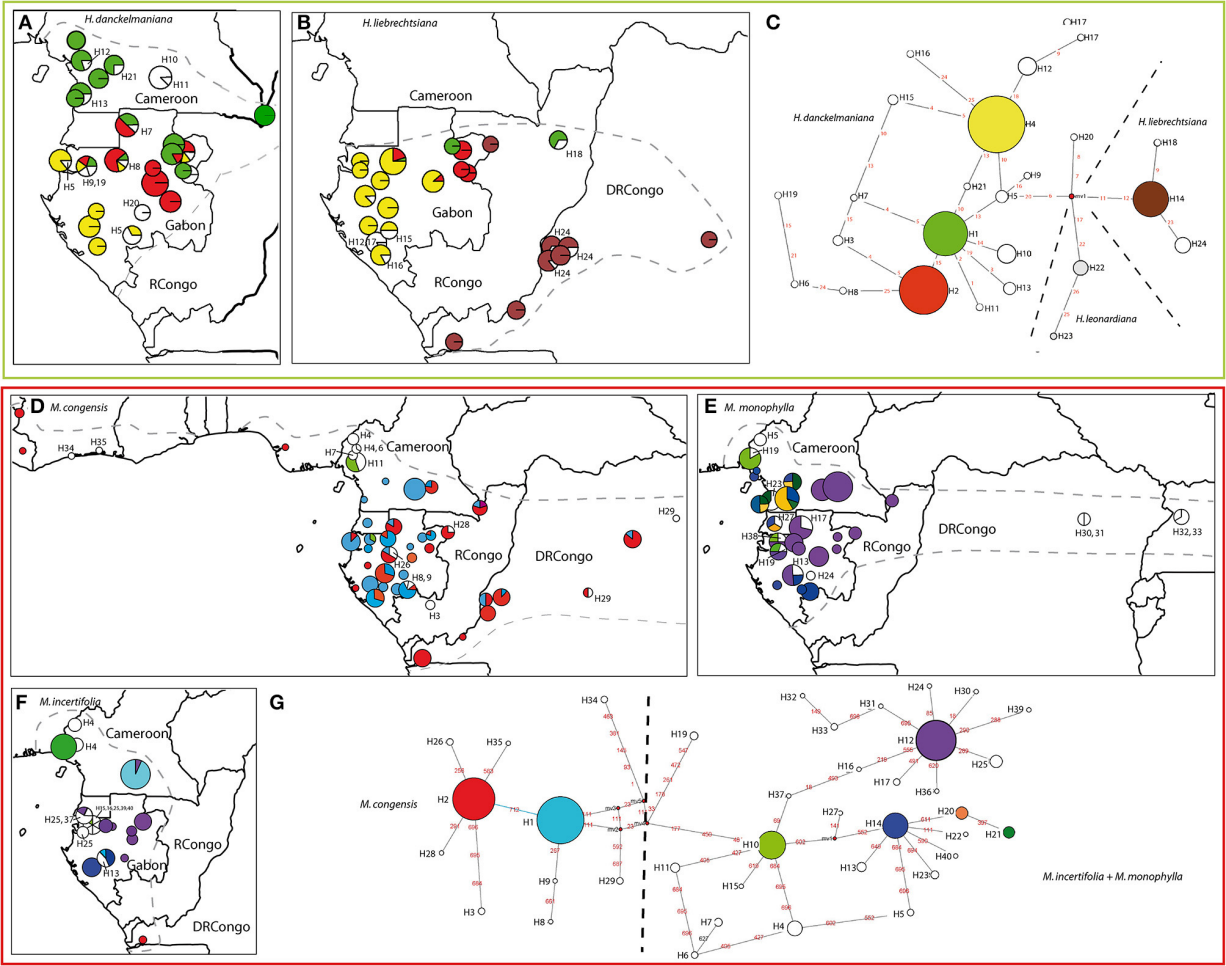
**Geographic distribution of chloroplast haplotypes and generic haplotype networks based on *trnC-petN1r* updated from Ley and Hardy (2010, 2014)**. *Haumania danckelmaniana* **(A, C)**, *H. liebrechtsiana* **(B, C)**, *Marantochloa congensis* **(D, G)**, *M. monophylla* **(E, G)**, *M. incertifolia* **(F, G)**. Gray hatched line: species distribution range. Sizes of circles are proportional to sample sizes at each locality in the geographic maps and proportional to haplotype frequency in the haplotype network. Frequent haplotypes are color-coded, rare haplotypes are number-coded. Stippled lines throughout the networks delineate groups of haplotypes according to the species in which they are usually found, but haplotypes can also be shared among species. Red numbers along branches are IDs of mutations, mv indicate median vectors (Bandelt et al., 1999).

Haplotype networks based on *trnC-petN1r* required 11–27(32) mutations (without torso) within species and 11–38(56) mutations within genera. Haplotypes did never show a high divergence neither within nor between congeneric species (Figures 2, 3). Within species haplotypes differed from the closest other haplotype generally by one mutation. Only few exceptions presented a distance of up to three mutations between closest haplotypes within species. All species networks included loops except for *Halopegia azurea, Haumania liebrechtsiana* and *Marantochloa incertifolia*. *Marantochloa monophylla* was the only species that showed two intraspecific divergent lineages. Between species, maximum distances between nearest haplotypes ranged between two to three mutations. In all networks we found a few individuals that belonged to one morphological species but exhibited the same haplotypes as individuals from the other morphological species.

### Phylogeographic patterns within each genus and species

*Halopegia azurea* was the species with the lowest haplotype diversity (11 haplotypes) resulting in a simple network without loops (Figure 2B). The only frequent haplotype was distributed over the whole Lower Guinean-Congolian range of the species (Figure 2A). Localities with additional one to several rare geographically restricted haplotypes divergent by one mutation from the single widespread haplotype were found around the Cameroonian Volcanic Line and the Chaillu Massif in Gabon. In West Africa three divergent haplotypes were found. They were most closely related (different by three mutations) to the rare haplotype of the Cameroonian Volcanic Line.

The two species from the genus *Megaphrynium* presented very different phylogeographic patterns. In *Mega. trichogynum* (Figure 2D) there was one widespread haplotype covering the whole distribution area of the species and another frequent haplotype restricted to Gabon. The diversity center in this species was found in the North of Gabon where the frequent haplotypes overlapped in their distribution and three rare haplotypes also occurred. *Mega. macrostachyum* presented four haplotypes (H1, 2, 9, 16) exclusive to different, large geographic areas (Southwest Cameroon, Southwest Gabon to DRCongo (Bas Congo), North to Northwest Gabon, East Gabon/East Cameroon/Congos, Figure 2C). Each widespread haplotype was co-occurring with closely related and geographically restricted haplotypes. This resulted in five areas of increased haplotype diversity: the Cameroonian volcanic line, western DRCongo, northern Gabon, coastal northwestern Gabon (near Libreville) and the Cristal Mountains area in Gabon. Only five *Megaphrynium* individuals out of 282 carried a haplotype typical of the other species.

The spatial genetic structure of species from the genera *Haumania* and *Marantochloa* were already discussed in previous publications (Ley and Hardy, 2010, 2014) but updated here (Figure 3). *Haumania danckelmaniana* (Figure 3A) exhibited three haplotypes each covering a different large geographic area (Cameroon + northern Gabon, eastern Gabon, western Gabon). Additionally, there were several geographically very restricted haplotypes in localities found almost all over the species' distribution range. *H. liebrechtsiana* (Figure 3B) carried the same haplotypes as *H. danckelmaniana* in Gabon where both species occur in sympatry. In the Congo basin *H. liebrechtsiana* carried specific haplotypes: one widespread haplotype occurring from the Atlantic coast in DRCongo to the Center of the Congo Basin and several rare haplotypes being concentrated at the middle course of the Congo river.

In the genus *Marantochloa* there were two distinct patterns when comparing species. *M. congensis* (Figure 3D), the most widespread species, had two widespread haplotypes found across its entire distribution range and a few rare (locally restricted) haplotypes concentrated along the coast of Ivory Coast, in the Cameroonian Volcanic Line, in East DRCongo and in a corridor from the southern Chaillu Massif in Gabon to eastern Cameroon. *M. monophylla* (Figure 3E) in contrast exhibited a strong geographic pattern of two genetically distinct haplogroups, one distributed along the Atlantic coast, the other one east of that toward the Congo Basin. Major diversity centers were found in mountain ranges: Cameroonian Volcanic Line and Ngovayang (Cameroon); Cristal Mountains and the southern Chaillu Massif (Gabon); and in the Albertine Rift Valley (Uganda) (for localities compare Figure 1). Whereas, *M. congensis* and *M. monophylla* did hardly share any haplotypes (only one at Mount Cameroon), *M. incertifolia* (Figure 3F) shared half of its haplotypes with either one or the other sister species, *M. congensis* and *M. monophylla*. In western Cameroon and the Cristal Mountains there was one haplotype (H10) shared between all three *Marantochloa* species occurring there.

The fixation indices (*G*_ST_: 0.15–0.77 and *N*_ST_: 0.14–0.83, Table 4) were higher in *Haumania liebrechtsiana, Marantochloa incertifolia* and *Megaphrynium macrostachyum* and rather low in *Megaphrynium trichogynum*. Fixation indices varied somewhat according to grid cell size but the ranking of species was generally fairly consistent. There was always a marked difference in *G*_ST_ and *N*_ST_ between congeners: *Mega. macrostachyum > Mega. trichogynum; H. liebrechtsiana > H. danckelmaniana; M. incertifolia > M. monophylla > M. congensis*. In most species a marginally significant to very significant phylogeographic signal (*N*_ST_ > *G*_ST_) could be detected at least at one scale (0.75°, 1.5°, and/or 3°) (Table 4). The signal was most clear in *M. congensis* and *M. monophylla*. No phylogeographic signal could be detected in *Halopegia azurea, Haumania liebrechtsiana* and *Megaphrynium trichogynum*.

**Table 4:** **Global *G*_ST_ and *N*_ST_ for the eight study species in Lower Guinea**.

**Species**	**Total Number of grid cells**			**0.75°**		**1.5°**		**3°**	
	**0.75° all(>3indiv)**	**1.5° all(>3indiv)**	**3° all(>3indiv)**	*G*_**ST**_	*N*_**ST**_	*G*_**ST**_	*N*_**ST**_	*G*_**ST**_	*N*_**ST**_
HalAzu	39 (18)	24 (16)	12 (10)	0.66	0.60	0.53	0.45	0.47	0.34
HauDanck	25 (19)	15 (12)	7 (7)	0.54	0.60	0.54	0.65^*^	0.58	0.60
HauLieb	24 (15)	12 (9)	9 (6)	0.67	0.70	0.58	0.65	0.59	0.67
MarCong	42 (21)	24 (18)	11 (10)	0.52	0.64^*^	0.57	0.83^**^	0.42	0.71^*^
MarIncert	16 (9)	9 (8)	9 (7)	0.75	0.82^(*)^	0.72	0.77^(*)^	0.77	0.83
MarMono	32 (18)	19 (12)	11 (7)	0.58	0.70^(*)^	0.54	0.71^**^	0.42	0.47
MegaMacro	39 (22)	23 (17)	11 (11)	0.60	0.69^*^	0.58	0.68^*^	0.62	0.68
MegaTrich	33 (21)	15 (12)	9 (8)	0.33	0.26	0.26	0.24	0.15	0.14

Test of significance for N_ST_ > G_ST_:

(*) , marginally significant (p < 0.1);

* , significant (p < 0.05);

** , highly significant (p < 0.01). All the G_ST_ values were significantly higher than 0 (p < 0.001). For abbreviations of species names see Table 1.

### Congruence of genetic diversity pattern among species

As general geographic patterns of diversity were independent of grid cell size, only results based on 0.75° grid cells are reported here. Standardized effective numbers of alleles, gene diversity and phylogenetic diversity per grid cell and species revealed a significant geographic effect according to the ANOVA analyses (*NAe*: *F* = 1.79, *P* = 0.01; *He*: *F* = 2.59, *P* < 0.001; *v*: *F* = 12.64, *P* < 0.001). By contrast, the ANOVA test was non-significant for the mean frequency of endemic haplotypes (*End*; *F* = 0.99, *P* = 0.49) and the genetic distinctiveness per grid cell *(S'i; F* = 0.86, *P* = 0.69; for 1.5°: *F* = 1.47, *P* = 0.12). Averaged standardized effective numbers of haplotypes per cell across species showed that diversity is highest in Cristal Mountains (Gabon) followed by northern and southern Gabon and Cameroonian volcanic line (Supplementary Figure 3, 4). By contrast south-western and eastern Gabon and coastal western and eastern Cameroon displayed below-average diversity values. South-western Cameroon and the DRCongo displayed close to average values. The multi-species pattern for phylogenetic diversity (*v*) was similar (Supplementary Figures 3, 4).

**Figure 4:**
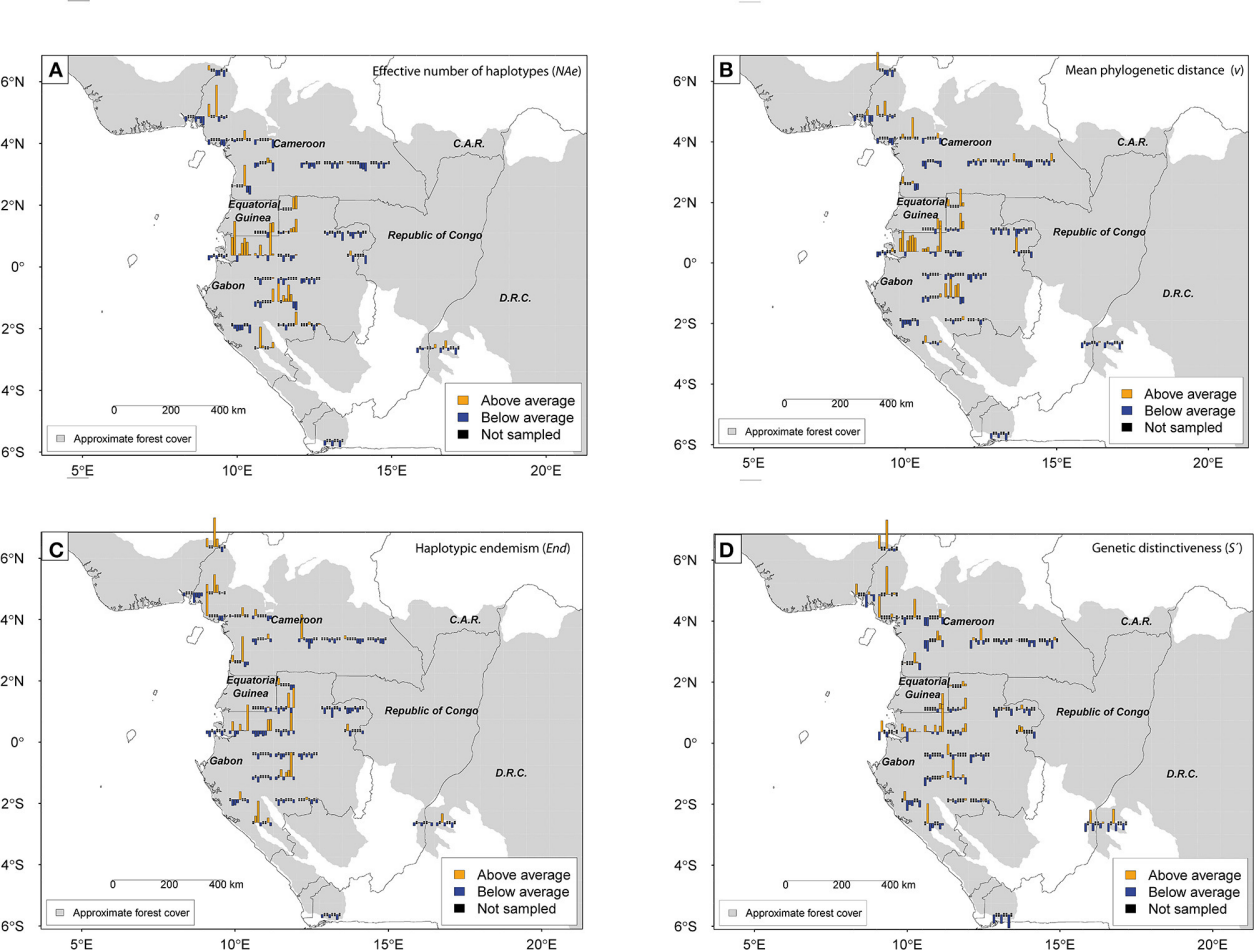
**Geographic distribution of standardized (i.e., centered and reduced) genetic diversity and endemism for eight Marantaceae species in Lower Guinea for grid cell size 0.75°**. Effective number of haplotypes (*NAe*) **(A)**; mean phylogenetic distance between individuals (*v*) **(B)**; haplotypic endemism (haplotype range <200 km, *End*) **(C)**; Genetic distinctiveness of each grid cell (*S'_i_*) **(D)**. Distinctiveness above or below average is based on standardized pairwise genetic distance (*S'_kij_* computed for each species) among populations where genetic distance is estimated as the number of mutational steps between two individuals drawn from two populations (*v_ij_*). Species along barplots from left to right are: *Halopegia azurea, Haumania danckelmaniana, H. liebrechtsiana, Marantochloa congensis, M. incertifolia, M. monophylla, Megaphrynium macrostachyum* and *Mega. trichogynum*.

Comparing diversity patterns pairwise between species, the Pearson correlation tests revealed congruence between *Halopegia azurea, Haumania danckelmaniana, M. incertifolia, M. congensis* and *M. monophylla* (Table 5, Supplementary Table 5) with two main common centers of diversity in Gabon: the western Cristal Mountains area close to Libreville and the northern Chaillu Massif (Figure 4, Supplementary Tables 6, 7). Diversity centers of *M. congensis* are beside the Cameroonian volcanic line in Cameroon, the Cristal Mountains area, the northern Gabon and only observed in this species: the southern Chaillu Massif of Gabon and the northern part of the RCongo (see Figure 4, Supplementary Tables 6, 7). Furthermore, *Mega. macrostachyum* and *Mega. trichogynum* were inter-correlated for *NAe*. These two species showed many centers of genetic diversity well distributed across Cameroon and Gabon. They shared the center of diversity in the southwest of Gabon with *H. liebrechtsiana* and *M. congensis*. Concerning pattern of endemism there is congruence between *Marantochloa monophylla* and *Haumania liebrechtsiana* and between *M. monophylla* and *M. incertifolia* (the latter only at 0.75°) as well as between *Halopegia azurea* and *M. congensis* and between *Halopegia azurea* and *H. danckelmaniana* (but the latter only detectable at 1.5° grid because not enough shared cells at 0.75°). Interestingly, there was no congruence in the patterns of haplotypic endemism across species (Supplementary Table 8).

**Table 5:** **Pearson correlation of the effective number of haplotypes (*NAe*, lower diagonal) and within-cell phylogenetic diversity (*v*, upper diagonal) between species for grid cell size of 0.75°**.

	**HaloAzu**	**HauDanck**	**HauLieb**	**MarCong**	**MarIncert**	**MarMono**	**MegaMacro**	**MegaTrich**
HaloAzu		0.93^**^	0.63	0.42	0.18	0.69^*^	0.04	0.05
HauDanck	0.95^**^		−0.07	0.85^**^	0.97^**^	0.62^(*)^	0.30	0.04
HauLieb	0.13	−0.30		−0.12	0.44	0.45	−0.33	−0.04
MarCong	0.51^(*)^	0.47	0.43		0.34	0.76^*^	0.30	−0.09
MarIncert	0.79^*^	0.95^*^	0.17	−0.05		0.71	0.52	0.03
MarMono	0.76^*^	0.55	−0.15	0.59^(*)^	0.81^(*)^		0.01	−0.33
MegaMacro	0.20	0.25	0.06	0.01	0.17	−0.03		0.39
MegaTrich	−0.12	0.05	0.14	−0.10	0.32	−0.25	0.53^(*)^	

(*) , marginally significant (p < 0.1);

* , significant (p < 0.05);

** , highly significant (p < 0.01). For abbreviations of species names see Table 1.

### Congruence of genetic distinctiveness patterns among species

The genetic distinctiveness per grid cell for each species is presented in Figure 4D. Above average levels of population distinctiveness for three or more species are reached in the Cameroonian volcanic line and in north-western Gabon (Libreville/coastal Gabon and western Cristal Mountains). In contrast, South and East Gabon, East Cameroon and DRCongo displayed always low levels of distinctiveness for most species.

There were only very few species pairs that showed statistically significant congruent patterns of genetic distinctiveness among grid cells (*S'_ij_*); there were three at grid cell size 0.75° (Table 6, *Megaphrynium macrostachyum* with *Marantochloa congensis* and *Marantochloa monophylla*, and *Marantochloa monophylla* with *Marantochloa congensis*) and three at grid cell size 1.5° (Supplementary Table 9).

**Table 6:** **Results of Mantel test comparing pairwise standardized distinctiveness among grid cells (*S*'_*ij*_) between species pairs for grid cell size of 0.75°**.

	**HaloAzu**	**HauDanck**	**HauLieb**	**MarCong**	**MarIncert**	**MarMono**	**MegaMacro**	**MegaTrich**
HaloAzu		7	7	12	7	9	14	9
HauDanck	0.15		6	10	5	9	9	12
HauLieb	−0.23	0		9	4	5	7	10
MarCong	0.4	−0.22	−0.39		7	10	12	9
MarIncert	−0.31	−0.02	−0.05	−0.23		5	6	6
MarMono	0.08	0.14	0.02	0.35^*^	−0.44		12	11
MegaMacro	−0.03	−0.21	−0.11	0.6^**^	0.26	0.4^*^		14
MegaTrich	0.17	0.09	−0.07	−0.1	0.21	0.1	0.1	

Upper diagonal: number of grid cells shared between species; lower diagonal: correlation coefficient. Significant values (p < 0.05) and highly significant values (p < 0.01) are written in bold and indicated by

* and

** , respectively. For abbreviations of species names see Table 1.

## Discussion

In this study, phylogeographic patterns of the plastid genome of eight herb and liana species from the family Marantaceae were compared in Lower Guinea. We expected that profound vegetation changes might have left their imprints in the distribution pattern of genetic diversity of species, and that similar species responses would lead to congruent phylogeographic patterns. In our study, however, we did not find overall congruence in the pattern of genetic diversity, endemism and distinctiveness across all study species but rather multiple patterns characteristic for one or a few species. Thus, there was not a uniform congruence of genetic pattern with the putative rainforest refugia proposed by Maley (1996). Our results indicate either idiosyncratic histories of the chosen taxa, or that once congruent genetic patterns resulting from similar species responses to particular climatic changes are already overlain by younger historical events (Alexandre et al., 1998; Maley and Brenac, 1998; Maley, 2002) leaving new individual imprints in the genetic patterns of species. Here, compared to tree species, phylogeographic patterns in herbs might reflect younger evolutionary events due to their shorter life cycles (for life cycles in perennial herbs/lianas see Putz, 1990; Gerwing, 2004; Brandes et al., 2011).

### Genetic diversity and differentiation between sister species in lower guinea

In the eight Marantaceae species studied, the level of genetic diversity at the plastid gene sequenced (7–19 haplotypes per species, see also nucleotide diversity) was similar to that found for the same plastid marker in tree species from Lower Guinea (6–24 haplotypes per species, (Dauby et al., 2014a); for nucleotide diversity see Heuertz et al., 2013). The high molecular diversity found in *Marantochloa monophylla* was congruent with its high morphological diversity – an exceptional morphological and genetic diversity (*NAe, v*) was found in Ngovayang Mountain in Cameroon. By contrast, genetic diversity was especially low in *Halopegia azurea*, a selfing species (Ley and Claßen-Bockhoff, 2013). Although selfing should not *per se* affect the diversity of maternally inherited genomes, it might enhance selective sweeps by generating a global linkage between nuclear and cytoplasmic genomes (Glemin et al., 2006), a possible explanation for the low diversity observed in the plastid genome.

The divergence of haplotypes within and between Marantaceae sister species was rather low (1–2 mutations) indicating a low degree of interspecific molecular divergence, potentially due to relatively recent speciation events. Species seem not to have yet established strong species boundaries which was probably the reason for the observation of recurrent hybridization events in sympatric regions in almost all sister species pairs considered here (see also Ley and Hardy, 2014).

### Spatial genetic structure and phylogeographic signal within species

A spatial genetic structure was found in all species (see significant *G*_ST_ values) indicating intra-specific population differentiation. In addition, a significant phylogeographic pattern (*N*_ST_ > *G*_ST_) could be detected in five of the eight Marantaceae species. This implies that, for these species at least, some of their populations have evolved in isolation for long enough to generate related haplotypes that tend to co-occur locally. Such phylogeographic pattern is expected if species survived in multiple isolated refugia. Only *Halopegia azurea, Haumania liebrechtsiana* and *Megaphrynium trichogynum* do not show such a signal. In all three species the low number of haplotypes (<10) prevents sufficient testing power.

Genetic differentiation between areas (*G*_ST_) in the eight Marantaceae species were comparable to values found in maternally inherited markers in many other angiosperm taxa, including tropical African trees (see Duminil et al., 2007; Dauby et al., 2014a). This is in contrast to our expectation of more sub-structuring in (perennial) herbs/lianas than in trees and may indicate rather similar dispersal and population structure in both growth form groups.

Within the Marantaceae, *G*_ST_ values seem to correlate superficially with dispersal ability (see also Petit et al., 2003): the *G*_ST_ was lowest in *Megaphrynium trichogynum* whose red fleshy fruits are ape/monkey dispersed (Williamson et al., 1990; Tutin and Fernandez, 1993; White and Abernethy, 1997), in *Halopegia azurea* (dispersal mode still unknown) and in *M. congensis* (see interpretation below), while it was highest in species of *Haumania liebrechtsiana* with large, probably gravity dispersed fruits, and in *Marantochloa incertifolia* with rather isolated occurrences and an extremely low production of flowers and fruits (5–15 flowers per inflorescence flowering sparsely over a month with a fruit set of 3–6%, see Ley, 2008; Ley and Claßen-Bockhoff, 2013). Fruits are here dispersed by small birds and/or water (Tutin, 1998; Ley, 2008). A rather low *G*_ST_ value in the Marantaceae is presented by *Mega. trichogynum* (*G*_ST_ = 0.33). However, there is no indication that its fruits are better dispersed than the ones of its sister species *Mega. macrostachyum* (fruit morphology compare in Dhetchuvi, 1996) which presents a much higher *G*_ST_ value. There are other contrasting *G*_ST_ values between sister species pairs (Table 4). However, we see a possible explanation in terms of dispersal ability difference only for the lower *G*_ST_ value found in *M. congensis* compared to its sister species. The three investigated *Marantochloa* species produce rather small amounts of fruits (see Ley and Claßen-Bockhoff, 2013). *M. congensis* is the only species which additionally frequently propagates by vegetative means, producing large quantities of bulbils which might be dispersed by water and animals, potentially contributing to an efficient gene flow between populations and a rapid clonal expansion of the species distribution range (Kennedy, 2000; Ley, 2008). *M. congensis* is the species with the largest current distribution range of the three investigated *Marantochloa* species, occurring from West Africa to eastern DRCongo (Dhetchuvi, 1996). The observation of a shared, possibly ancestral haplotype in the Cameroonian Volcanic Line and Cristal Mountains might suggest that the three species originated there. We favor ancestral haplotype over chloroplast capture, as we are dealing here with a haplotype in the center of the haplotype network between the three species. Under this assumption, the much larger distribution range of *M. congensis* might be explained by better dispersal capacities.

### Concordance of observed genetic patterns across species and with postulated pleistocene refugia in lower guinea

We demonstrated congruent geographic patterns of diversity across species: local genetic diversity is congruently high for six out of eight study species in the Cristal Mountains area and low for seven out of eight study species in eastern Cameroon, eastern and southern coastal Gabon, and Bas Congo (Mayumbe) in DRCongo. By contrast, values of above-average frequency of endemic haplotypes can almost be found in every grid cell when taking all species together. However, few species pairs display correlated patterns of endemic haplotype frequencies. Similarly, there is no general correlation in distinctiveness indices among Marantaceae species, while such a correlation was reported among five of eight tree species for Lower Guinea (Dauby et al., 2014a).

To interpret these patterns, it is worth noting that endemic haplotypes are potentially the best indicators for refuge areas in general (stable populations are expected to accumulate endemic haplotypes that have not the opportunity to emigrate), and distinctiveness indices might be best indicators of refuge areas that are not source of adjacent areas. High diversity is expected in refuge areas with historically large population sizes (but not under small but stable population size), but also in areas recolonized from multiple differentiated populations (secondary contact zones), a situation where phylogenetic diversity (*v*) should peak (Petit et al., 2003). As we found interspecific congruence in genetic diversity but not in endemism and distinctiveness, the data do not support a hypothesis whereby the different Marantaceae species would have primarily survived in the same set of refugia during periods of climate deterioration. Nevertheless, the correlation in diversity indices might indicate that there are some shared secondary contact and/or refuge areas. In fact, some high diversity areas, like the Cristal Mountains, might have been both a refuge for some species and a secondary contact zone for other species, or even the two for some species (if we imagine that a refuge area becomes “invaded” by an expanding population from another origin).

Overall, we found marked differences in patterns of haplotype distribution across species: (i) Some species are characterized by mostly parapatric distributions of their frequent haplotypes (*H. danckelmaniana, Mega. macrostachyum* and *M. monophylla*; plus *H. liebrechtsiana* though in this case the pattern may be due to plastid capture). Here a common pattern becomes apparent distinguishing Cameroon from south-western Gabon and eastern Gabon. Frequent haplotypes often overlap in their distribution range in the Mount Cristal area. Each of these individual distribution ranges overlaps with a different postulated refugium allowing two different scenarios: either an expansion of each of the frequent haplotypes from a central refugium in the Cristal Mountains area into different directions to Cameroon, south-western Gabon and eastern Gabon, or the other way round with an expansion from three different refugia with an overlap today in the Cristal Mountains area. A similar pattern of restricted haplotype distribution ranges was also found in some tree species and has here been attributed to the retraction of these species to different refugia. In trees it might additionally have been coupled with an adaptation to different climatic conditions evoked by the East-West rainfall gradient and the North-South seasonal gradient in Lower Guinea (Duminil et al., 2013; Heuertz et al., 2013). (ii) A second haplotype distribution pattern is characterized by a wide distribution of one or a few frequent haplotypes over the entire distribution range of a species (*Halopegia azurea, M. congensis, Mega. trichogynum*). These species might have a single locality of origin from where an expansion took place across the current distribution range (though a second refugium but without visible expansion would explain the endemic haplotypes found in the Cameroonian Volcanic Line for *M. congensis*). So far the detected diversity and endemism pattern in these three species suggest a refugium within Lower Guinea based on high diversity and endemism e.g., Cristal Mountains with or without Chaillu Massif and/or Cameroonian Volcanic Line and/or eastern Cameroon depending on species. The only study species so far showing evidence of a refugium outside Lower Guinea in DRCongo is *H. liebrechtsiana* (see also Ley and Hardy, 2010, 2014). As already found in trees (see Hardy et al., 2013) major rivers seem not to play an important role as barriers to gene flow in Marantaceae in contrast to evidence found in animals (e.g., Gonder and Disotell, 2006; Anthony et al., 2007; Nicolas et al., 2011).

There is repeatedly genetic evidence in species for the Cristal Mountains being a refuge area (see Koffi et al., 2011; Dauby et al., 2014b). The fact that in the Marantaceae the high diversity of the Cristal Mountains area is not associated with high endemism for at least half of the species suggests that the high diversity is best explained by a recolonization from several sources, rather than by a refuge effect, at least for these species. Note however that an area might be a refuge and at the same time have been “invaded” by other sources.

The Cameroonian volcanic line is a locality well-known for its high species diversity and endemism level, which has been interpreted as a signature of a past forest refuge (see Sosef, 1994; Maley, 1996). In the Marantaceae, only species with large distribution ranges from Lower Guinea to West Africa present high genetic diversity and/or distinctiveness and/or endemism values here. This is in accordance with patterns found in widespread trees (see also Lowe et al., 2010) and might be due to a refuge effect, i.e., accumulation of mutations in stable populations, and/or to a topographical effect, i.e., differentiation between geographically close populations isolated by mountainous barriers (see also Dauby et al., 2014a).

Assuming that the limited concordance between phylogeographic patterns of Marantaceae species in Lower Guinea reflects their idiosyncratic histories of past population fragmentation, one may question the relative importance of chance (species survived by chance in one or several refugia following forest fragmentation) and ecological adaptations (e.g., species survived only in refugia reflecting the optimum of their climatic tolerance). As all species are currently co-occurring in all potential refugia without a marked adaptation to different habitats and/or climate regimes (see Dhetchuvi, 1996) we favor the hypothesis that demographic stochasticity affecting population survival as well as rare long distance dispersal driving recolonization routes played a major role in the resulting phylogeographic patterns. For tree species, congruence of genetic distinctiveness patterns was observed in northern Lower Guinea but not in southern Lower Guinea (Dauby et al., 2014a). This pattern was tentatively explained by a less drastic forest cover reduction in southern Lower Guinea where multiple micro-refugia (e.g., gallery forests) would have remained (see Kingdon, 1980; Dupont et al., 2000; Leal, 2001). The high ecological drift associated with these micro-refugia would imply that each one would have hosted a limited number of typical rainforest species, which might have led to the observed idiosyncratic demographic histories of species. This hypothesis might also hold for our Marantaceae species.

A current limitation for the interpretation of our data is the difficulty to date population divergence or admixture and provide a confirmation that such events are concomitant with Pleistocene climate changes and not earlier or later events of climate change. Plastid markers are not ideal for this purpose due to their relatively low mutation rate. Additional studies based on nuclear sequencing should bring new insights.

### Beyond lower guinea—the role of upper guinea and congolia

Assessing the importance of the areas adjacent to the East and the West of Lower Guinea (Congolia and Upper Guinea, respectively) for speciation and population differentiation is still difficult due to a lack of sufficient data. Patterns so far documented indicate that these areas have widespread haplotypes but also endemic haplotypes. In Upper Guinea several refugia were postulated by Maley (1996, see Figure 1) and the dry Dahomey gap in Benin might play an important role in isolating Upper and Lower Guinea (see Hardy et al., 2013), although the two forest blocks were probably connected during the Humid Holocene period (c. 6–9 kr BP). This can explain why several species are still restricted to Western Africa today (White, 1979; for Marantaceae see Schnell, 1957; Jongkind, 2008) and endemic haplotypes are found there (e.g., *Halopegia azurea*, see also Duminil et al., 2013) advocating the uniqueness of this area. Similarly, the Congo Basin and the adjacent eastern mountain range are interesting areas. Preliminary data suggest overall genetic diversity to be low in this area for most species, defining this region rather as an area of expansion. Some authors have suggested that Marantaceae species could have been spread to east Cameroon/RCongo due to human activities (Maley, 2001; Brncic et al., 2009). However, despite a rather fragmentary sampling, endemic haplotypes have also been detected in the Congo Basin and in the Albertine Rift Valley (see *M. monophylla*). This suggests a rather long existence of those species in that area.

## Author contributions

Alexandra C. Ley has been conducting research on Marantaceae since her PhD starting in 2004. She did most of the field collections and genetic laboratory manipulations, analyzed the chloroplast haplotype distribution and diversity pattern and took a lead in the editing of the article. Gilles Dauby developed the analyses of divergence of haplotype distribution across species in a previous project and applied his knowledge here on the Marantaceae dataset. Julia Köhler and Catherina Wypior conducted a “Forschungsgruppenpraktikum” on the acquisition and analyses of the genetic data of one species of *Megaphrynium* each. Martin Röser and Olivier J. Hardy are both group leaders. The latter conducted the analyses of spatial genetic pattern and endemism and significantly contributed to the discussion of results. All co-authors gave their final approval of the version to be published.

### Conflict of interest statement

The authors declare that the research was conducted in the absence of any commercial or financial relationships that could be construed as a potential conflict of interest.
