# Supplementary material for: Comparative phylogeography of eight herbs and lianas (Marantaceae) in central African rainforests
Source: Front Genet. 2014 Nov 19;5:403. doi: 10.3389/fgene.2014.00403 (PMC4237126; doi:10.3389/fgene.2014.00403)
Supplement: Supplementary file 1 [file DataSheet1.PDF]

Supplementary Material

Comparative phylogeography of eight herbs and lianas (Marantaceae) in central African rainforests

A.C. Ley[1,2*], G. Dauby[2], J. Köhler[1], C. Wypior[1], M. Röser[1] and O.J. Hardy[2]

[1] Institut für Geobotanik und Botanischer Garten, University Halle-Wittenberg, Halle (Saale), Germany.
[2]Evolutionary Biology and Ecology, Faculté des Sciences, Université Libre de Bruxelles, Brussels, Belgium.

*** Correspondence:** Dr. Alexandra C. Ley, Institut für Geobotanik und Botanischer Garten, University Halle-Wittenberg
Neuwerk 21, 06108 Halle (Saale), Germany
Alexandra.Ley@botanik.uni-halle.de

Supplementary Data

1. Supplementary Figures and Tables

1.1. Suplementary Tables

Supplementary Table 1. Genbank accession numbers (Acc. Nb.).

Taxon	Sample ID	Haplotype	Genetic Marker	Acc. Nb.
Halopegia				
Halopegia azurea	ACL0439	H1	*trnC-petN1R*	KM243931
Halopegia azurea	ACL0447	H2	*trnC-petN1R*	KM24393
Halopegia azurea	ACL0480	H3	*trnC-petN1R*	KM243933
Halopegia azurea	ACL0586	H4	*trnC-petN1R*	KM243934
Halopegia azurea	ACL11650	H5	*trnC-petN1R*	KM376052
Halopegia azurea	ACL11702	H6	*trnC-petN1R*	KM376053
Halopegia azurea	ACL11726	H7	*trnC-petN1R*	KM376054
Halopegia azurea	ACL11758	H8	*trnC-petN1R*	KM376055
Halopegia azurea	ACL5058	H9	*trnC-petN1R*	KM376056
Halopegia azurea	ACL5654	H10	*trnC-petN1R*	KM376057
Halopegia azurea	ACL8644	H11	*trnC-petN1R*	KM243935
Haumania				
Haumania danckelmaniana	ACL0033	Hap1	*trnC-petN1R*	KM376088
Haumania danckelmaniana	ACL0035	Hap2	*trnC-petN1R*	KM376089
Haumania danckelmaniana	ACL0039	Hap3	*trnC-petN1R*	KM376090

Haumania danckelmaniana	ACL0046	Hap4	*trnC-petN1R*	KM376091
Haumania danckelmaniana	ACL0049	Hap5	*trnC-petN1R*	KM376092
Haumania danckelmaniana	ACL0651	Hap6	*trnC-petN1R*	KM376093
Haumania danckelmaniana	ACL0721	Hap7	*trnC-petN1R*	KM376094
Haumania danckelmaniana	ACL0834	Hap8	*trnC-petN1R*	KM376095
Haumania danckelmaniana	ACL0857	Hap9	*trnC-petN1R*	KM376096
Haumania danckelmaniana	ACL0949	Hap10	*trnC-petN1R*	KM376097
Haumania danckelmaniana	ACL0992	Hap11	*trnC-petN1R*	KM376098
Haumania danckelmaniana	ACL4206	Hap17	*trnC-petN1R*	KM376099
Haumania danckelmaniana	ACL4213	Hap18	*trnC-petN1R*	KM376100
Haumania danckelmaniana	ACL4425	Hap19	*trnC-petN1R*	KM376101
Haumania liebrechtsiana	ACL1056	Hap1	*trnC-petN1R*	KM376065
Haumania liebrechtsiana	ACL1043	Hap2	*trnC-petN1R*	KM376066
Haumania liebrechtsiana	ACL1023	Hap4	*trnC-petN1R*	KM376067
Haumania liebrechtsiana	ACL1112	Hap10	*trnC-petN1R*	KM376068
Haumania liebrechtsiana	ACL10755	Hap12	*trnC-petN1R*	KM376069
Haumania liebrechtsiana	ACL1084	Hap13	*trnC-petN1R*	KM376070
Haumania liebrechtsiana	ACL1088	Hap14	*trnC-petN1R*	KM376071
Haumania liebrechtsiana	ACL1118	Hap15	*trnC-petN1R*	KM376072
Haumania liebrechtsiana	ACL1216	Hap16	*trnC-petN1R*	KM376073
Haumania liebrechtsiana	ACL8917	Hap21	*trnC-petN1R*	KM376074
Marantochloa				
Marantochloa congensis	ACL0001	Hap1	*trnC-petN1R*	KM376102
Marantochloa congensis	ACL0003	Hap2	*trnC-petN1R*	KM376103
Marantochloa congensis	ACL11722	Hap3	*trnC-petN1R*	KM376104
Marantochloa congensis	ACL11768	Hap4	*trnC-petN1R*	KM376105
Marantochloa congensis	ACL11769	Hap6	*trnC-petN1R*	KM376106
Marantochloa congensis	ACL11788	Hap7	*trnC-petN1R*	KM376107
Marantochloa congensis	ACL2025	Hap8	*trnC-petN1R*	KM376108
Marantochloa congensis	ACL2032	Hap9	*trnC-petN1R*	KM376109
Marantochloa congensis	ACL2108	Hap10	*trnC-petN1R*	KM376110
Marantochloa congensis	ACL2120	Hap11	*trnC-petN1R*	KM376111
Marantochloa congensis	ACL7153	Hap12	*trnC-petN1R*	KM376112
Marantochloa congensis	ACL4530	Hap28	*trnC-petN1R*	KM376113
Marantochloa congensis	ACL4535	Hap29	*trnC-petN1R*	KM376114
Marantochloa congensis	ACL7125	Hap34	*trnC-petN1R*	KM376115
Marantochloa congensis	ACL7131	Hap35	*trnC-petN1R*	KM376116
Marantochloa congensis	ACL0002	H1	*psbA-trnH2*	KM872091
Marantochloa congensis	ACL0004	H2	*psbA-trnH2*	KM872092
Marantochloa congensis	ACL0009	H3	*psbA-trnH2*	KM872093
Marantochloa congensis	ACL2032	H4	*psbA-trnH2*	KM872094
Marantochloa congensis	ACL2106	H5	*psbA-trnH2*	KM872095
Marantochloa congensis	ACL4234	H6	*psbA-trnH2*	KM872097
Marantochloa congensis	ACL4534	H7	*psbA-trnH2*	KM872096
Marantochloa incertifolia	ACL2350	Hap1	*trnC-petN1R*	KM376075
Marantochloa incertifolia	ACL11740	Hap4	*trnC-petN1R*	KM376076
Marantochloa incertifolia	ACL2414	Hap10	*trnC-petN1R*	KM376077

Marantochloa incertifolia	ACL2344	Hap12	*trnC-petN1R*	KM376078
Marantochloa incertifolia	ACL2351	Hap13	*trnC-petN1R*	KM376079
Marantochloa incertifolia	ACL2353	Hap14	*trnC-petN1R*	KM376080
Marantochloa incertifolia	ACL2412	Hap15	*trnC-petN1R*	KM376081
Marantochloa incertifolia	ACL2413	Hap16	*trnC-petN1R*	KM376082
Marantochloa incertifolia	ACL4386	Hap25	*trnC-petN1R*	KM376083
Marantochloa incertifolia	ACL7469	Hap37	*trnC-petN1R*	KM376084
Marantochloa incertifolia	ACL7785	Hap39	*trnC-petN1R*	KM376085
Marantochloa incertifolia	ACL7894	Hap40	*trnC-petN1R*	KM376086
Marantochloa incertifolia	ACL7167	Hap2	*trnC-petN1R*	KM376087
Marantochloa monophylla	ACL11764	Hap5	*trnC-petN1R*	KM376133
Marantochloa monophylla	ACL2735	Hap10	*trnC-petN1R*	KM376134
Marantochloa monophylla	ACL2536	Hap12	*trnC-petN1R*	KM376135
Marantochloa monophylla	ACL2549	Hap13	*trnC-petN1R*	KM376136
Marantochloa monophylla	ACL2582	Hap14	*trnC-petN1R*	KM376137
Marantochloa monophylla	ACL2548	Hap17	*trnC-petN1R*	KM376138
Marantochloa monophylla	ACL2752	Hap19	*trnC-petN1R*	KM376139
Marantochloa monophylla	ACL2786	Hap20	*trnC-petN1R*	KM376140
Marantochloa monophylla	ACL2789	Hap21	*trnC-petN1R*	KM376141
Marantochloa monophylla	ACL2869	Hap22	*trnC-petN1R*	KM376142
Marantochloa monophylla	ACL2883	Hap23	*trnC-petN1R*	KM376143
Marantochloa monophylla	ACL4223	Hap24	*trnC-petN1R*	KM376144
Marantochloa monophylla	ACL4526	Hap27	*trnC-petN1R*	KM376145
Marantochloa monophylla	ACL4649	Hap30	*trnC-petN1R*	KM376146
Marantochloa monophylla	ACL4650	Hap31	*trnC-petN1R*	KM376147
Marantochloa monophylla	ACL4653	Hap32	*trnC-petN1R*	KM376148
Marantochloa monophylla	ACL4654	Hap33	*trnC-petN1R*	KM376149
Marantochloa monophylla	ACL7490	Hap38	*trnC-petN1R*	KM376150
Megaphrynium				
Megaphrynium macrostachyum	ACL10129	Hap1	*trnC-petN1R*	KM376117
Megaphrynium macrostachyum	ACL10182	Hap2	*trnC-petN1R*	KM376118
Megaphrynium macrostachyum	ACL11346_JFG252	Hap3	*trnC-petN1R*	KM376119
Megaphrynium macrostachyum	ACL10531	Hap4	*trnC-petN1R*	KM376120
Megaphrynium macrostachyum	ACL11004	Hap6	*trnC-petN1R*	KM376121
Megaphrynium macrostachyum	ACL11235	Hap7	*trnC-petN1R*	KM376122
Megaphrynium macrostachyum	ACL11799	Hap8	*trnC-petN1R*	KM376123
Megaphrynium macrostachyum	ACL11802	Hap9	*trnC-petN1R*	KM376124
Megaphrynium macrostachyum	ACL11813	Hap10	*trnC-petN1R*	KM376125
Megaphrynium macrostachyum	ACL3015	Hap11	*trnC-petN1R*	KM376126
Megaphrynium macrostachyum	ACL3069	Hap13	*trnC-petN1R*	KM376127
Megaphrynium macrostachyum	ACL3119	Hap14	*trnC-petN1R*	KM376128
Megaphrynium macrostachyum	ACL3128	Hap15	*trnC-petN1R*	KM376129
Megaphrynium macrostachyum	ACL3186	Hap16	*trnC-petN1R*	KM376130
Megaphrynium macrostachyum	ACL3201	Hap17	*trnC-petN1R*	KM376131
Megaphrynium macrostachyum	ACL8191	Hap23	*trnC-petN1R*	KM376132
Megaphrynium trichogynum	ACL10206	Hap3	*trnC-petN1R*	KM376058
Megaphrynium trichogynum	ACL10745	Hap5	*trnC-petN1R*	KM376059

Megaphrynium trichogynum	ACL3285	Hap18	*trnC-petN1R*	KM376060
Megaphrynium trichogynum	ACL3305	Hap19	*trnC-petN1R*	KM376061
Megaphrynium trichogynum	ACL3335	Hap20	*trnC-petN1R*	KM376062
Megaphrynium trichogynum	ACL3442	Hap21	*trnC-petN1R*	KM376063
Megaphrynium trichogynum	ACL3471	Hap22	*trnC-petN1R*	KM376064

Supplementary Table 2. List of grid cells under 0.75° (ID), coordinates (center of cell) and number of individuals sampled for each species. Only cells from Lower Guinea (between 8° and 18° longitude E) containing at least 3 samples for at least one species were considered. For abbreviations of species names see Table 1. Lat, Latitude; Long, Longitude. For position of grid cells see S.2A.

ID	Lat	Long	HaloAzu	HauDanck	HauLieb	MarCong	MarIncert	MarMono	MegaMacro	MegaTricho	Total
M11x16	4.875	8.625	3	0	0	0	20	6	3	4	36
M12x10	0.375	9.375	3	7	0	10	0	0	6	0	26
M12x15	4.125	9.375	6	0	0	0	0	3	5	0	14
M12x16	4.875	9.375	8	0	0	15	3	0	12	0	38
M12x18	6.375	9.375	3	0	0	3	3	0	3	0	12
M13x10	0.375	10.125	8	5	3	3	12	8	6	5	50
M13x13	2.625	10.125	0	9	0	0	0	9	3	5	26
M13x15	4.125	10.125	0	7	0	0	0	3	0	0	10
M13x7	-1.875	10.125	0	6	3	6	6	0	0	3	24
M13x9	-0.375	10.125	0	0	7	0	0	0	0	0	7
M14x10	0.375	10.875	5	8	16	5	4	4	9	10	61
M14x11	1.125	10.875	0	0	0	0	0	3	5	8	16
M14x14	3.375	10.875	6	4	0	0	0	10	6	3	29
M14x15	4.125	10.875	3	3	0	0	0	0	3	3	12
M14x6	-2.625	10.875	0	3	13	7	0	0	3	7	33
M14x8	-1.125	10.875	4	0	4	0	0	0	0	4	12
M14x9	-0.375	10.875	0	0	3	0	0	0	0	5	8
M15x10	0.375	11.625	0	0	4	0	0	3	7	5	19
M15x11	1.125	11.625	0	5	0	4	0	4	4	6	23
M15x12	1.875	11.625	0	3	0	0	0	0	3	6	12
M15x7	-1.875	11.625	0	3	0	3	0	0	0	4	10
M15x8	-1.125	11.625	7	0	4	9	5	7	6	7	45
M15x9	-0.375	11.625	5	0	6	6	0	0	0	0	17

M16x14	3.375	12.375	3	5	0	8	15	3	0	0	34
M16x7	-1.875	12.375	0	0	0	11	0	6	0	5	22
M16x9	-0.375	12.375	3	15	0	12	0	5	0	0	35
M17x11	1.125	13.125	0	12	0	0	5	0	0	5	22
M17x14	3.375	13.125	0	0	0	3	0	3	0	0	6
M17x2	-5.625	13.125	4	0	0	7	0	0	5	0	16
M17x8	-1.125	13.125	0	6	0	0	0	0	0	0	6
M18x10	0.375	13.875	0	3	4	0	0	0	0	3	10
M18x11	1.125	13.875	0	7	4	6	0	3	0	0	20
M18x14	3.375	13.875	11	0	0	0	0	12	13	11	47
M19x14	3.375	14.625	0	0	0	3	0	3	5	0	11
M21x11	1.125	16.125	0	0	0	0	0	0	3	0	3
M21x12	1.875	16.125	0	4	0	0	0	0	0	0	4
M21x13	2.625	16.125	0	0	0	3	0	0	0	0	3
M21x6	-2.625	16.125	4	0	10	9	0	0	22	4	49
M22x11	1.125	16.875	0	0	3	0	0	0	0	0	3
M22x6	-2.625	16.875	5	0	14	8	0	0	8	0	35

Supplementary Table 3. List of grid cells under 1.5° (ID), coordinates (center of cell) and number of individuals sampled for each Marantaceae species. Only cells from Lower Guinea (between 8° and 18° longitude E) containing at least 3 samples for at least one species were considered for further analyses. For abbreviations of species names see Table 1. Lat, Latitude; Long, Longitude. For position of grid cells see S.2B.

ID	Lat	Long	HaloAzu	HauDanck	HauLieb	MarCong	MarIncert	MarMono	MegaMacro	MegaTricho	Total
M10x3	-2.65	16.45	9	0	24	17	0	0	30	4	84
M10x6	1.85	16.45	0	4	3	4	0	0	5	0	16
M5x5	0.35	8.95	3	7	0	10	0	0	6	2	28
M5x7	3.35	8.95	6	0	0	0	0	3	5	1	15
M5x8	4.85	8.95	8	0	0	10	23	6	14	5	66
M5x9	6.35	8.95	3	0	0	3	3	0	3	0	12
M6x3	-2.65	10.45	2	3	13	7	0	0	3	7	35
M6x4	-1.15	10.45	5	7	16	11	6	2	3	10	60
M6x5	0.35	10.45	12	7	15	5	15	12	18	27	111
M6x6	1.85	10.45	0	0	0	2	0	3	0	0	5
M6x7	3.35	10.45	11	21	0	2	0	23	12	12	81
M6x8	4.85	10.45	3	2	0	5	0	0	2	0	12
M7x3	-2.65	11.95	3	3	0	14	0	8	1	8	37
M7x4	-1.15	11.95	8	1	5	11	6	8	7	8	54
M7x5	0.35	11.95	12	23	19	25	6	11	16	8	120
M7x6	1.85	11.95	3	8	0	6	0	6	7	12	42
M8x1	-5.65	13.45	4	0	2	7	1	0	5	1	20
M8x4	-1.15	13.45	0	6	0	0	0	0	0	0	6
M8x5	0.35	13.45	5	22	9	10	6	4	4	12	72
M8x7	3.35	13.45	15	7	0	12	15	18	15	14	96
M9x2	-4.15	14.95	0	0	3	1	0	0	0	0	4
M9x5	0.35	14.95	1	0	1	4	0	2	1	0	9
M9x7	3.35	14.95	2	0	0	3	0	3	5	0	13
			115	121	110	169	81	109	162	131	998

Supplementary Table 4. Within cell diversity pattern at the *trnC-petN1r* region in eight Afrotropical Marantaceae species in Lower Guinea for grid cell size 1.5° (average across grid cells, [range]). N, number; *NAe*, Effective N alleles (Nielsen et al., 2003); He, gene diversity corrected for sample size (Nei 1978); v, mean phylogenetic distance between individuals (Pons & Petit 1996); End, mean proportion of individuals carrying endemic alleles. For abbreviations of species names see Table 1.

			mean [range] per cell of				
**Species**	**Total sample size**	**N cells with >2 samples**	**N samples**	**NAe**	**He**	**v**	**End**
HaloAzu	110	16	6.88	1.54	0.25	0.33	0.10
			[3,15]	[1,3.04]	[0,0.68]	[0,1.33]	[0,1]
HauDanck	118	12	9.83	1.90	0.38	0.62	0.16
			[3,23]	[1,5.44]	[0,0.86]	[0,1.9]	[0,0.86]
HauLieb	107	9	11.89	1.55	0.34	1.26	0.16
			[3,24]	[1,2.55]	[0,0.67]	[0,4]	[0,0.54]
MarCongc	164	18	9.11	1.57	0.30	0.49	0.13
			[3,25]	[1,4.18]	[0,0.78]	[0,2.47]	[0,1]
MarIncert	80	8	10.00	2.04	0.25	1.31	0.45
			[3,23]	[1,5.23]	[0,0.87]	[0,4.93]	[0,1]
MarMono	105	12	8.75	1.99	0.34	1.31	0.13
			[3,23]	[1,5.18]	[0,1]	[0,4.43]	[0,0.43]
MegaMacro	158	17	9.29	1.81	0.36	0.97	0.15
			[3,30]	[1,4.53]	[0,0.78]	[0,2.67]	[0,0.86]
MegaTricho	127	12	10.58	1.59	0.32	0.39	0.02
			[4,27]	[1,2.58]	[0,0.62]	[0,1.26]	[0,0.08]

Supplementary Table 5. Pearson correlation of the effective number of haplotypes (*NAe*, lower diagonal) and within-cell phylogenetic diversity (*v*, upper diagonal) between species for grid cell size of 1.5°. (*), marginally significant (p < 0.5); *, significant (p < 0.1); **, highly significant (p < 0.01). For abbreviations of species names see Table 1.

	HaloAzu	HauDanck	HauLieb	MarCong	MarIncert	MarMono	MegaMacro	MegaTrich
HaloAzu		0.27	0.43	0.38	0.25	0.75*	-0.06	0.24
HauDanck	0.55		-0.49	0.76*	0.86(*)	0.88**	0.44	0.68*
HauLieb	-0.17	-0.42		-0.17	0.71	0.57	0.13	-0.32
MarCong	0.38	0.15	0.48		0.24	0.61(*)	0.21	0.19
MarIncert	0.78*	0.96**	-0.14	-0.03		0.86*	0.29	0.41
MarMono	0.23	0.21	-0.15	0.03	0.98**		0.01	0.27
MegaMacro	0.35	0.69	-0.16	0.07	0.34	0.13		0.61*
MegaTrich	0.10	0.64	-0.11	-0.41	0.47	-0.05	0.45	

Supplementary Table 6. Haplotypic endemism (*End*), distinctiveness (*S´*), effective number of haplotypes (*NAe*) and (phylo)diversity (*v*), Number individuals (Nb) per species for each grid cell (0.75° grid cell size). NA, no data available. For the geographic positions of grid cells (ID) see S.2A.

				Halopegia azurea			
**ID**	**Lat**	**Long**	**Nb**	***NAe***	***v***	***End***	***S´***
M11x16	4.875	8.625	3	-0.579	-0.647	-0.374	0.666
M12x10	0.375	9.375	3	-0.579	-0.647	-0.374	-0.524
M12x15	4.125	9.375	6	-0.579	-0.647	3.728	1.344
M12x16	4.875	9.375	8	1.435	1.196	0.139	-0.064
M12x18	6.375	9.375	3	0.562	2.112	0.994	0.849
M13x10	0.375	10.125	8	2.121	1.584	-0.374	0.497
M13x13	2.625	10.125	0	NA	NA	NA	NA
M13x15	4.125	10.125	0	NA	NA	NA	NA
M13x7	-1.875	10.125	0	NA	NA	NA	NA
M13x9	-0.375	10.125	0	NA	NA	NA	NA
M14x10	0.375	10.875	5	0.223	0.346	-0.374	0.390
M14x11	1.125	10.875	0	NA	NA	NA	NA
M14x14	3.375	10.875	6	-0.579	-0.647	-0.374	-0.524
M14x15	4.125	10.875	3	-0.579	-0.647	-0.374	-0.524
M14x6	-2.625	10.875	0	NA	NA	NA	NA
M14x8	-1.125	10.875	4	-0.579	-0.647	-0.374	-0.524
M14x9	-0.375	10.875	0	NA	NA	NA	NA
M15x10	0.375	11.625	0	NA	NA	NA	NA
M15x11	1.125	11.625	0	NA	NA	NA	NA
M15x12	1.875	11.625	0	NA	NA	NA	NA
M15x7	-1.875	11.625	0	NA	NA	NA	NA
M15x8	-1.125	11.625	7	2.455	1.633	-0.374	0.379
M15x9	-0.375	11.625	5	-0.579	-0.647	-0.374	0.666
M16x14	3.375	12.375	3	-0.579	-0.647	-0.374	-0.524
M16x7	-1.875	12.375	0	NA	NA	NA	NA
M16x9	-0.375	12.375	3	-0.579	-0.647	-0.374	-0.524
M17x11	1.125	13.125	0	NA	NA	NA	NA
M17x14	3.375	13.125	0	NA	NA	NA	NA
M17x2	-5.625	13.125	4	-0.579	-0.647	-0.374	-0.524
M17x8	-1.125	13.125	0	NA	NA	NA	NA
M18x10	0.375	13.875	0	NA	NA	NA	NA
M18x11	1.125	13.875	0	NA	NA	NA	NA
M18x14	3.375	13.875	11	0.153	0.892	0.372	-0.007
M19x14	3.375	14.625	0	NA	NA	NA	NA
M21x11	1.125	16.125	0	NA	NA	NA	NA
M21x12	1.875	16.125	0	NA	NA	NA	NA
M21x13	2.625	16.125	0	NA	NA	NA	NA
M21x6	-2.625	16.125	4	-0.579	-0.647	-0.374	-0.524
M22x11	1.125	16.875	0	NA	NA	NA	NA
M22x6	-2.625	16.875	5	-0.579	-0.647	-0.374	-0.524

					Haumania danckelmanniana		
**ID**	**Lat**	**Long**	**Nb**	***NAe***	***v***	***End***	***S´***
M11x16	4.875	8.625	0	NA	NA	NA	NA
M12x10	0.375	9.375	7	-0.272	-0.463	-0.709	0.693
M12x15	4.125	9.375	0	NA	NA	NA	NA
M12x16	4.875	9.375	0	NA	NA	NA	NA
M12x18	6.375	9.375	0	NA	NA	NA	NA
M13x10	0.375	10.125	5	4.002	2.547	1.081	0.313
M13x13	2.625	10.125	9	0.011	0.817	0.783	0.033
M13x15	4.125	10.125	7	-0.272	0.510	-0.070	-0.472
M13x7	-1.875	10.125	6	-0.457	-0.949	-0.709	0.570
M13x9	-0.375	10.125	0	NA	NA	NA	NA
M14x10	0.375	10.875	8	-0.300	0.355	-0.709	0.030
M14x11	1.125	10.875	0	NA	NA	NA	NA
M14x14	3.375	10.875	4	-0.457	-0.949	-0.709	-0.705
M14x15	4.125	10.875	3	-0.061	-0.066	0.783	-0.546
M14x6	-2.625	10.875	3	-0.061	0.817	0.783	1.239
M14x8	-1.125	10.875	0	NA	NA	NA	NA
M14x9	-0.375	10.875	0	NA	NA	NA	NA
M15x10	0.375	11.625	0	NA	NA	NA	NA
M15x11	1.125	11.625	5	-0.178	-0.313	-0.709	-0.231
M15x12	1.875	11.625	3	-0.061	0.817	0.783	0.061
M15x7	-1.875	11.625	3	-0.061	-0.066	-0.709	0.047
M15x8	-1.125	11.625	0	NA	NA	NA	NA
M15x9	-0.375	11.625	0	NA	NA	NA	NA
M16x14	3.375	12.375	5	-0.178	-0.313	2.871	0.172
M16x7	-1.875	12.375	0	NA	NA	NA	NA
M16x9	-0.375	12.375	15	-0.457	-0.949	-0.709	-0.150
M17x11	1.125	13.125	12	-0.360	-0.646	-0.709	-0.526
M17x14	3.375	13.125	0	NA	NA	NA	NA
M17x2	-5.625	13.125	0	NA	NA	NA	NA
M17x8	-1.125	13.125	6	-0.457	-0.949	-0.709	-0.217
M18x10	0.375	13.875	3	0.534	1.699	0.783	0.400
M18x11	1.125	13.875	7	-0.457	-0.949	-0.709	-0.049
M18x14	3.375	13.875	0	NA	NA	NA	NA
M19x14	3.375	14.625	0	NA	NA	NA	NA
M21x11	1.125	16.125	0	NA	NA	NA	NA
M21x12	1.875	16.125	4	-0.457	-0.949	-0.709	-0.664
M21x13	2.625	16.125	0	NA	NA	NA	NA
M21x6	-2.625	16.125	0	NA	NA	NA	NA
M22x11	1.125	16.875	0	NA	NA	NA	NA
M22x6	-2.625	16.875	0	NA	NA	NA	NA

					Haumania liebrechtsiana		
**ID**	**Lat**	**Long**	**Nb**	***NAe***	***v***	***End***	***S´***
M11x16	4.875	8.625	0	NA	NA	NA	NA
M12x10	0.375	9.375	0	NA	NA	NA	NA
M12x15	4.125	9.375	0	NA	NA	NA	NA

M12x16	4.875	9.375	0	NA	NA	NA	NA
M12x18	6.375	9.375	0	NA	NA	NA	NA
M13x10	0.375	10.125	3	-0.742	-0.556	-0.656	-0.618
M13x13	2.625	10.125	0	NA	NA	NA	NA
M13x15	4.125	10.125	0	NA	NA	NA	NA
M13x7	-1.875	10.125	3	-0.742	-0.556	-0.656	-0.646
M13x9	-0.375	10.125	7	0.029	-0.120	0.196	-0.261
M14x10	0.375	10.875	16	1.219	0.542	-0.656	0.015
M14x11	1.125	10.875	0	NA	NA	NA	NA
M14x14	3.375	10.875	0	NA	NA	NA	NA
M14x15	4.125	10.875	0	NA	NA	NA	NA
M14x6	-2.625	10.875	13	2.447	0.013	2.554	-0.479
M14x8	-1.125	10.875	4	-0.742	-0.556	-0.656	-0.262
M14x9	-0.375	10.875	3	-0.742	-0.556	-0.656	-0.273
M15x10	0.375	11.625	4	-0.742	-0.556	-0.656	-0.268
M15x11	1.125	11.625	0	NA	NA	NA	NA
M15x12	1.875	11.625	0	NA	NA	NA	NA
M15x7	-1.875	11.625	0	NA	NA	NA	NA
M15x8	-1.125	11.625	4	0.730	2.780	0.834	1.203
M15x9	-0.375	11.625	6	-0.742	-0.556	-0.656	-0.298
M16x14	3.375	12.375	0	NA	NA	NA	NA
M16x7	-1.875	12.375	0	NA	NA	NA	NA
M16x9	-0.375	12.375	0	NA	NA	NA	NA
M17x11	1.125	13.125	0	NA	NA	NA	NA
M17x14	3.375	13.125	0	NA	NA	NA	NA
M17x2	-5.625	13.125	0	NA	NA	NA	NA
M17x8	-1.125	13.125	0	NA	NA	NA	NA
M18x10	0.375	13.875	4	-0.742	-0.556	-0.656	0.314
M18x11	1.125	13.875	4	-0.742	-0.556	-0.656	0.253
M18x14	3.375	13.875	0	NA	NA	NA	NA
M19x14	3.375	14.625	0	NA	NA	NA	NA
M21x11	1.125	16.125	0	NA	NA	NA	NA
M21x12	1.875	16.125	0	NA	NA	NA	NA
M21x13	2.625	16.125	0	NA	NA	NA	NA
M21x6	-2.625	16.125	10	-0.241	-0.395	-0.060	0.835
M22x11	1.125	16.875	3	0.907	1.817	1.331	-0.412
M22x6	-2.625	16.875	14	0.843	-0.192	1.047	0.897

					Marantochloa congensis		
**ID**	**Lat**	**Long**	**Nb**	***NAe***	***v***	***End***	***S´***
M11x16	4.875	8.625	0	NA	NA	NA	NA
M12x10	0.375	9.375	10	-0.399	-0.376	-0.398	-0.328
M12x15	4.125	9.375	0	NA	NA	NA	NA
M12x16	4.875	9.375	15	3.690	1.728	2.157	1.767
M12x18	6.375	9.375	3	-0.661	-0.525	3.434	1.818
M13x10	0.375	10.125	3	0.200	1.316	-0.398	0.229
M13x13	2.625	10.125	0	NA	NA	NA	NA
M13x15	4.125	10.125	0	NA	NA	NA	NA

M13x7	-1.875	10.125	6	-0.661	-0.525	-0.398	-0.267
M13x9	-0.375	10.125	0	NA	NA	NA	NA
M14x10	0.375	10.875	5	-0.056	-0.260	-0.398	-0.133
M14x11	1.125	10.875	0	NA	NA	NA	NA
M14x14	3.375	10.875	0	NA	NA	NA	NA
M14x15	4.125	10.875	0	NA	NA	NA	NA
M14x6	-2.625	10.875	7	0.232	-0.187	-0.398	-0.288
M14x8	-1.125	10.875	0	NA	NA	NA	NA
M14x9	-0.375	10.875	0	NA	NA	NA	NA
M15x10	0.375	11.625	0	NA	NA	NA	NA
M15x11	1.125	11.625	4	0.107	-0.214	-0.398	-0.126
M15x12	1.875	11.625	0	NA	NA	NA	NA
M15x7	-1.875	11.625	3	-0.661	-0.525	-0.398	-0.195
M15x8	-1.125	11.625	9	0.357	-0.157	-0.398	-0.127
M15x9	-0.375	11.625	6	-0.175	-0.295	-0.398	-0.046
M16x14	3.375	12.375	8	-0.661	-0.525	-0.398	-0.268
M16x7	-1.875	12.375	11	0.340	-0.005	0.299	-0.112
M16x9	-0.375	12.375	12	-0.661	-0.525	-0.398	-0.070
M17x11	1.125	13.125	0	NA	NA	NA	NA
M17x14	3.375	13.125	3	-0.661	-0.525	-0.398	-0.265
M17x2	-5.625	13.125	7	-0.661	-0.525	-0.398	-0.549
M17x8	-1.125	13.125	0	NA	NA	NA	NA
M18x10	0.375	13.875	0	NA	NA	NA	NA
M18x11	1.125	13.875	6	-0.175	-0.295	-0.398	-0.254
M18x14	3.375	13.875	0	NA	NA	NA	NA
M19x14	3.375	14.625	3	-0.661	-0.525	-0.398	-0.327
M21x11	1.125	16.125	0	NA	NA	NA	NA
M21x12	1.875	16.125	0	NA	NA	NA	NA
M21x13	2.625	16.125	3	1.490	3.524	0.879	0.511
M21x6	-2.625	16.125	9	-0.007	-0.238	-0.398	-0.476
M22x11	1.125	16.875	0	NA	NA	NA	NA
M22x6	-2.625	16.875	8	-0.319	-0.344	-0.398	-0.494

				Marantochloa incertifolia			
**ID**	**Lat**	**Long**	**Nb**	***NAe***	***v***	***End***	***S´***
M11x16	4.875	8.625	20	-0.610	-0.690	-1.163	-0.825
M12x10	0.375	9.375	0	NA	NA	NA	NA
M12x15	4.125	9.375	0	NA	NA	NA	NA
M12x16	4.875	9.375	3	-0.610	-0.690	0.944	-0.012
M12x18	6.375	9.375	3	-0.610	-0.690	0.944	-0.130
M13x10	0.375	10.125	12	1.436	1.883	0.769	0.061
M13x13	2.625	10.125	0	NA	NA	NA	NA
M13x15	4.125	10.125	0	NA	NA	NA	NA
M13x7	-1.875	10.125	6	-0.610	-0.690	0.944	-0.341
M13x9	-0.375	10.125	0	NA	NA	NA	NA
M14x10	0.375	10.875	4	0.105	-0.111	-0.636	0.419
M14x11	1.125	10.875	0	NA	NA	NA	NA
M14x14	3.375	10.875	0	NA	NA	NA	NA

M14x15	4.125	10.875	0	NA	NA	NA	NA
M14x6	-2.625	10.875	0	NA	NA	NA	NA
M14x8	-1.125	10.875	0	NA	NA	NA	NA
M14x9	-0.375	10.875	0	NA	NA	NA	NA
M15x10	0.375	11.625	0	NA	NA	NA	NA
M15x11	1.125	11.625	0	NA	NA	NA	NA
M15x12	1.875	11.625	0	NA	NA	NA	NA
M15x7	-1.875	11.625	0	NA	NA	NA	NA
M15x8	-1.125	11.625	5	1.964	1.409	0.523	0.025
M15x9	-0.375	11.625	0	NA	NA	NA	NA
M16x14	3.375	12.375	15	-0.457	0.270	-1.163	0.733
M16x7	-1.875	12.375	0	NA	NA	NA	NA
M16x9	-0.375	12.375	0	NA	NA	NA	NA
M17x11	1.125	13.125	5	-0.610	-0.690	-1.163	0.071
M17x14	3.375	13.125	0	NA	NA	NA	NA
M17x2	-5.625	13.125	0	NA	NA	NA	NA
M17x8	-1.125	13.125	0	NA	NA	NA	NA
M18x10	0.375	13.875	0	NA	NA	NA	NA
M18x11	1.125	13.875	0	NA	NA	NA	NA
M18x14	3.375	13.875	0	NA	NA	NA	NA
M19x14	3.375	14.625	0	NA	NA	NA	NA
M21x11	1.125	16.125	0	NA	NA	NA	NA
M21x12	1.875	16.125	0	NA	NA	NA	NA
M21x13	2.625	16.125	0	NA	NA	NA	NA
M21x6	-2.625	16.125	0	NA	NA	NA	NA
M22x11	1.125	16.875	0	NA	NA	NA	NA
M22x6	-2.625	16.875	0	NA	NA	NA	NA

				Marantochloa monophylla			
**ID**	**Lat**	**Long**	**Nb**	***NAe***	***v***	***End***	***S´***
M11x16	4.875	8.625	6	-0.259	0.683	-0.536	0.175
M12x10	0.375	9.375	0	NA	NA	NA	NA
M12x15	4.125	9.375	3	-0.645	-0.587	-0.536	0.216
M12x16	4.875	9.375	0	NA	NA	NA	NA
M12x18	6.375	9.375	0	NA	NA	NA	NA
M13x10	0.375	10.125	8	2.023	1.967	0.032	0.159
M13x13	2.625	10.125	9	2.437	0.288	2.999	0.664
M13x15	4.125	10.125	3	1.062	2.461	0.979	1.155
M13x7	-1.875	10.125	0	NA	NA	NA	NA
M13x9	-0.375	10.125	0	NA	NA	NA	NA
M14x10	0.375	10.875	4	-0.645	-0.587	-0.536	-0.190
M14x11	1.125	10.875	3	-0.645	-0.587	-0.536	-0.210
M14x14	3.375	10.875	10	0.566	-0.244	-0.081	0.593
M14x15	4.125	10.875	0	NA	NA	NA	NA
M14x6	-2.625	10.875	0	NA	NA	NA	NA
M14x8	-1.125	10.875	0	NA	NA	NA	NA
M14x9	-0.375	10.875	0	NA	NA	NA	NA
M15x10	0.375	11.625	3	-0.645	-0.587	-0.536	-0.211

M15x11	1.125	11.625	4	0.422	-0.301	1.737	-0.071
M15x12	1.875	11.625	0	NA	NA	NA	NA
M15x7	-1.875	11.625	0	NA	NA	NA	NA
M15x8	-1.125	11.625	7	0.839	1.606	0.763	-0.126
M15x9	-0.375	11.625	0	NA	NA	NA	NA
M16x14	3.375	12.375	3	-0.645	-0.587	-0.536	-0.264
M16x7	-1.875	12.375	6	-0.645	-0.587	-0.536	0.106
M16x9	-0.375	12.375	5	-0.645	-0.587	-0.536	-0.378
M17x11	1.125	13.125	0	NA	NA	NA	NA
M17x14	3.375	13.125	3	-0.645	-0.587	-0.536	-0.262
M17x2	-5.625	13.125	0	NA	NA	NA	NA
M17x8	-1.125	13.125	0	NA	NA	NA	NA
M18x10	0.375	13.875	0	NA	NA	NA	NA
M18x11	1.125	13.875	3	-0.645	-0.587	-0.536	-0.423
M18x14	3.375	13.875	12	-0.645	-0.587	-0.536	-0.418
M19x14	3.375	14.625	3	-0.645	-0.587	-0.536	-0.516
M21x11	1.125	16.125	0	NA	NA	NA	NA
M21x12	1.875	16.125	0	NA	NA	NA	NA
M21x13	2.625	16.125	0	NA	NA	NA	NA
M21x6	-2.625	16.125	0	NA	NA	NA	NA
M22x11	1.125	16.875	0	NA	NA	NA	NA
M22x6	-2.625	16.875	0	NA	NA	NA	NA

				Megaphrynium macrostachyum			
**ID**	**Lat**	**Long**	**Nb**	***NAe***	***v***	***End***	***S´***
M11x16	4.875	8.625	3	-0.767	-0.829	-0.599	-0.085
M12x10	0.375	9.375	6	-0.284	0.380	-0.599	0.012
M12x15	4.125	9.375	5	-0.767	-0.829	-0.599	-0.015
M12x16	4.875	9.375	12	-0.279	-0.330	-0.020	0.112
M12x18	6.375	9.375	3	-0.767	-0.829	-0.599	-0.171
M13x10	0.375	10.125	6	1.551	1.648	-0.020	0.001
M13x13	2.625	10.125	3	-0.767	-0.829	-0.599	0.075
M13x15	4.125	10.125	0	NA	NA	NA	NA
M13x7	-1.875	10.125	0	NA	NA	NA	NA
M13x9	-0.375	10.125	0	NA	NA	NA	NA
M14x10	0.375	10.875	9	3.092	0.675	1.331	0.364
M14x11	1.125	10.875	5	1.052	1.259	0.096	0.346
M14x14	3.375	10.875	6	0.292	-0.346	0.559	0.296
M14x15	4.125	10.875	3	0.088	0.621	-0.599	0.509
M14x6	-2.625	10.875	3	0.088	-0.346	0.559	-0.377
M14x8	-1.125	10.875	0	NA	NA	NA	NA
M14x9	-0.375	10.875	0	NA	NA	NA	NA
M15x10	0.375	11.625	7	-0.368	-0.563	2.379	0.239
M15x11	1.125	11.625	4	0.569	1.890	-0.599	0.112
M15x12	1.875	11.625	3	1.370	2.071	-0.599	0.300
M15x7	-1.875	11.625	0	NA	NA	NA	NA
M15x8	-1.125	11.625	6	-0.767	-0.829	2.875	0.176
M15x9	-0.375	11.625	0	NA	NA	NA	NA

M16x14	3.375	12.375	0	NA	NA	NA	NA
M16x7	-1.875	12.375	0	NA	NA	NA	NA
M16x9	-0.375	12.375	0	NA	NA	NA	NA
M17x11	1.125	13.125	0	NA	NA	NA	NA
M17x14	3.375	13.125	0	NA	NA	NA	NA
M17x2	-5.625	13.125	5	-0.767	-0.829	-0.599	-0.831
M17x8	-1.125	13.125	0	NA	NA	NA	NA
M18x10	0.375	13.875	0	NA	NA	NA	NA
M18x11	1.125	13.875	0	NA	NA	NA	NA
M18x14	3.375	13.875	13	-0.767	-0.829	-0.599	-0.174
M19x14	3.375	14.625	5	-0.166	0.911	-0.599	0.196
M21x11	1.125	16.125	3	-0.767	-0.829	-0.599	-0.360
M21x12	1.875	16.125	0	NA	NA	NA	NA
M21x13	2.625	16.125	0	NA	NA	NA	NA
M21x6	-2.625	16.125	22	-0.102	-0.411	0.032	-0.308
M22x11	1.125	16.875	0	NA	NA	NA	NA
M22x6	-2.625	16.875	8	-0.767	-0.829	-0.599	-0.418

				Megaphrynium trichogynum			
**ID**	**Lat**	**Long**	**Nb**	***NAe***	***v***	***End***	***S´***
M11x16	4.875	8.625	4	-0.992	-0.728	-0.383	-0.427
M12x10	0.375	9.375	0	NA	NA	NA	NA
M12x15	4.125	9.375	0	NA	NA	NA	NA
M12x16	4.875	9.375	0	NA	NA	NA	NA
M12x18	6.375	9.375	0	NA	NA	NA	NA
M13x10	0.375	10.125	5	0.125	0.003	3.062	0.001
M13x13	2.625	10.125	5	-0.992	-0.728	-0.383	-0.427
M13x15	4.125	10.125	0	NA	NA	NA	NA
M13x7	-1.875	10.125	3	-0.992	-0.728	-0.383	-0.427
M13x9	-0.375	10.125	0	NA	NA	NA	NA
M14x10	0.375	10.875	10	0.171	3.701	1.340	2.430
M14x11	1.125	10.875	8	1.130	0.342	-0.383	0.283
M14x14	3.375	10.875	3	-0.992	-0.728	-0.383	-0.427
M14x15	4.125	10.875	3	-0.992	-0.728	-0.383	-0.427
M14x6	-2.625	10.875	7	0.656	0.204	-0.383	-0.124
M14x8	-1.125	10.875	4	1.490	0.414	-0.383	0.127
M14x9	-0.375	10.875	5	0.125	0.003	-0.383	-0.219
M15x10	0.375	11.625	5	0.125	0.003	-0.383	0.513
M15x11	1.125	11.625	6	1.535	0.921	2.488	0.692
M15x12	1.875	11.625	6	1.535	0.414	-0.383	0.127
M15x7	-1.875	11.625	4	1.490	0.414	-0.383	0.127
M15x8	-1.125	11.625	7	-0.992	-0.728	-0.383	-0.427
M15x9	-0.375	11.625	0	NA	NA	NA	NA
M16x14	3.375	12.375	0	NA	NA	NA	NA
M16x7	-1.875	12.375	5	0.125	0.003	-0.383	-0.219
M16x9	-0.375	12.375	0	NA	NA	NA	NA
M17x11	1.125	13.125	5	-0.992	-0.728	-0.383	-0.427
M17x14	3.375	13.125	0	NA	NA	NA	NA

M17x2	-5.625	13.125	0	NA	NA	NA	NA
M17x8	-1.125	13.125	0	NA	NA	NA	NA
M18x10	0.375	13.875	3	-0.992	-0.728	-0.383	-0.427
M18x11	1.125	13.875	0	NA	NA	NA	NA
M18x14	3.375	13.875	11	-0.992	-0.728	-0.383	-0.427
M19x14	3.375	14.625	0	NA	NA	NA	NA
M21x11	1.125	16.125	0	NA	NA	NA	NA
M21x12	1.875	16.125	0	NA	NA	NA	NA
M21x13	2.625	16.125	0	NA	NA	NA	NA
M21x6	-2.625	16.125	4	0.426	0.128	-0.383	0.102
M22x11	1.125	16.875	0	NA	NA	NA	NA
M22x6	-2.625	16.875	0	NA	NA	NA	NA

Supplementary Table 7. Haplotypic endemism (*End*), distinctiveness (*S´*), effective number of haplotypes (*NAe*) and phylogenetic diversity (*v*), Number of individuals (Nb) per species for each grid cell (1.5° grid cell size). NA, no data available. For the geographic positions of grid cells (ID) see S.2B.

Halopegia azurea

**ID**	**Lat**	**Long**	**Nb**	***NAe***	***v***	***End***	***S´***
M10x3	-2.65	16.45	9	-0.728	-0.792	-0.388	-0.550
M10x6	1.85	16.45	0	NA	NA	NA	NA
M5x5	0.35	8.95	3	-0.728	-0.792	-0.388	-0.550
M5x7	3.35	8.95	6	-0.728	-0.792	3.512	1.448
M5x8	4.85	8.95	8	1.190	1.172	0.100	0.556
M5x9	6.35	8.95	3	0.358	1.747	0.912	1.084
M6x3	-2.65	10.45	2	NA	NA	NA	NA
M6x4	-1.15	10.45	5	0.866	0.579	-0.388	-0.008
M6x5	0.35	10.45	12	2.049	1.707	-0.388	0.741
M6x6	1.85	10.45	0	NA	NA	NA	NA
M6x7	3.35	10.45	11	-0.728	-0.792	-0.388	-0.550
M6x8	4.85	10.45	3	-0.728	-0.792	-0.388	-0.550
M7x3	-2.65	11.95	3	-0.728	-0.792	-0.388	-0.550
M7x4	-1.15	11.95	8	1.843	1.172	-0.388	0.318
M7x5	0.35	11.95	12	0.513	0.477	-0.388	0.406
M7x6	1.85	11.95	3	-0.728	-0.792	-0.388	-0.550
M8x1	-5.65	13.45	4	-0.728	-0.792	-0.388	-0.550
M8x4	-1.15	13.45	0	NA	NA	NA	NA
M8x5	0.35	13.45	5	-0.728	-0.792	-0.388	-0.550
M8x7	3.35	13.45	15	-0.264	0.274	0.132	-0.143
M9x2	-4.15	14.95	0	NA	NA	NA	NA
M9x5	0.35	14.95	1	NA	NA	NA	NA
M9x7	3.35	14.95	2	NA	NA	NA	NA

Haumania danckelmaniana

**ID**	**Lat**	**Long**	**Nb**	***NAe***	***v***	***End***	***S´***
M10x3	-2.65	16.45	0	NA	NA	NA	NA

M10x6	1.85	16.45	4	-0.723	-1.052	-0.620	-0.556
M5x5	0.35	8.95	7	-0.424	-0.556	-0.620	0.176
M5x7	3.35	8.95	0	NA	NA	NA	NA
M5x8	4.85	8.95	0	NA	NA	NA	NA
M5x9	6.35	8.95	0	NA	NA	NA	NA
M6x3	-2.65	10.45	3	-0.083	0.749	0.696	0.908
M6x4	-1.15	10.45	7	-0.723	-1.052	-0.620	0.222
M6x5	0.35	10.45	7	2.832	2.255	0.508	-0.046
M6x6	1.85	10.45	0	NA	NA	NA	NA
M6x7	3.35	10.45	21	-0.162	0.657	0.320	-0.188
M6x8	4.85	10.45	2	NA	NA	NA	NA
M7x3	-2.65	11.95	3	-0.083	-0.152	-0.620	-0.150
M7x4	-1.15	11.95	1	NA	NA	NA	NA
M7x5	0.35	11.95	23	-0.560	-0.393	-0.620	-0.107
M7x6	1.85	11.95	8	0.791	0.847	-0.127	-0.162
M8x1	-5.65	13.45	0	NA	NA	NA	NA
M8x4	-1.15	13.45	6	-0.723	-1.052	-0.620	-0.039
M8x5	0.35	13.45	22	0.279	0.304	-0.441	-0.304
M8x7	3.35	13.45	7	-0.424	-0.556	2.764	0.245
M9x2	-4.15	14.95	0	NA	NA	NA	NA
M9x5	0.35	14.95	0	NA	NA	NA	NA
M9x7	3.35	14.95	0	NA	NA	NA	NA

				Haumania liebrechtsiana			
**ID**	**Lat**	**Long**	**Nb**	***NAe***	***v***	***End***	***S´***
M10x3	-2.65	16.45	24	-0.053	-0.579	0.279	0.420
M10x6	1.85	16.45	3	0.551	1.455	0.946	-0.682
M5x5	0.35	8.95	0	NA	NA	NA	NA
M5x7	3.35	8.95	0	NA	NA	NA	NA
M5x8	4.85	8.95	0	NA	NA	NA	NA
M5x9	6.35	8.95	0	NA	NA	NA	NA
M6x3	-2.65	10.45	13	2.173	-0.309	2.041	-0.372
M6x4	-1.15	10.45	16	-0.532	-0.485	-0.166	-0.240
M6x5	0.35	10.45	15	-0.855	-0.541	-0.833	-0.330
M6x6	1.85	10.45	0	NA	NA	NA	NA
M6x7	3.35	10.45	0	NA	NA	NA	NA
M6x8	4.85	10.45	0	NA	NA	NA	NA
M7x3	-2.65	11.95	0	NA	NA	NA	NA
M7x4	-1.15	11.95	5	0.035	1.919	0.234	0.882
M7x5	0.35	11.95	19	0.453	0.099	-0.833	-0.130
M7x6	1.85	11.95	0	NA	NA	NA	NA
M8x1	-5.65	13.45	2	NA	NA	NA	NA
M8x4	-1.15	13.45	0	NA	NA	NA	NA
M8x5	0.35	13.45	9	-0.587	-0.694	-0.833	0.251
M8x7	3.35	13.45	0	NA	NA	NA	NA
M9x2	-4.15	14.95	3	-1.185	-0.866	-0.833	0.202
M9x5	0.35	14.95	1	NA	NA	NA	NA
M9x7	3.35	14.95	0	NA	NA	NA	NA

Marantochloa congensis

ID	Lat	Long	Nb	***NAe***	***v***	***End***	***S´***
M10x3	-2.65	16.45	17	-0.180	-0.230	-0.413	-0.263
M10x6	1.85	16.45	4	0.196	-0.084	-0.413	-0.269
M5x5	0.35	8.95	10	-0.455	-0.421	-0.413	-0.305
M5x7	3.35	8.95	0	NA	NA	NA	NA
M5x8	4.85	8.95	10	3.594	3.106	2.691	1.735
M5x9	6.35	8.95	3	-0.790	-0.732	2.691	1.483
M6x3	-2.65	10.45	7	0.356	-0.026	-0.413	-0.298
M6x4	-1.15	10.45	11	-0.490	-0.446	-0.413	-0.305
M6x5	0.35	10.45	5	-0.014	2.034	-0.413	-0.133
M6x6	1.85	10.45	2	NA	NA	NA	NA
M6x7	3.35	10.45	2	NA	NA	NA	NA
M6x8	4.85	10.45	5	-0.790	-0.732	-0.413	0.707
M7x3	-2.65	11.95	14	0.097	0.150	0.031	-0.246
M7x4	-1.15	11.95	11	0.245	-0.046	-0.413	-0.298
M7x5	0.35	11.95	25	0.204	-0.035	-0.413	-0.271
M7x6	1.85	11.95	6	-0.167	-0.252	-0.413	-0.262
M8x1	-5.65	13.45	7	-0.790	-0.732	-0.413	-0.247
M8x4	-1.15	13.45	0	NA	NA	NA	NA
M8x5	0.35	13.45	10	0.369	-0.006	-0.413	-0.297
M8x7	3.35	13.45	12	-0.790	-0.732	-0.413	-0.308
M9x2	-4.15	14.95	1	NA	NA	NA	NA
M9x5	0.35	14.95	4	0.196	-0.084	0.363	-0.177
M9x7	3.35	14.95	3	-0.790	-0.732	-0.413	-0.247

Marantochloa incertifolia

ID	Lat	Long	Nb	***NAe***	***v***	***End***	***S´***
M10x3	-2.65	16.45	0	NA	NA	NA	NA
M10x6	1.85	16.45	0	NA	NA	NA	NA
M5x5	0.35	8.95	0	NA	NA	NA	NA
M5x7	3.35	8.95	0	NA	NA	NA	NA
M5x8	4.85	8.95	23	-0.407	-0.293	-0.693	-0.799
M5x9	6.35	8.95	3	-0.580	-0.710	1.195	0.131
M6x3	-2.65	10.45	0	NA	NA	NA	NA
M6x4	-1.15	10.45	6	-0.580	-0.710	1.195	-0.213
M6x5	0.35	10.45	15	1.435	1.273	0.761	0.044
M6x6	1.85	10.45	0	NA	NA	NA	NA
M6x7	3.35	10.45	0	NA	NA	NA	NA
M6x8	4.85	10.45	0	NA	NA	NA	NA
M7x3	-2.65	11.95	0	NA	NA	NA	NA
M7x4	-1.15	11.95	6	1.785	1.809	0.471	0.012
M7x5	0.35	11.95	6	-0.580	-0.710	-0.977	0.087
M7x6	1.85	11.95	0	NA	NA	NA	NA
M8x1	-5.65	13.45	1	NA	NA	NA	NA
M8x4	-1.15	13.45	0	NA	NA	NA	NA
M8x5	0.35	13.45	6	-0.580	-0.710	-0.977	-0.096

M8x7	3.35	13.45	15	-0.495	0.052	-0.977	0.833
M9x2	-4.15	14.95	0	NA	NA	NA	NA
M9x5	0.35	14.95	0	NA	NA	NA	NA
M9x7	3.35	14.95	0	NA	NA	NA	NA

Marantochloa monophylla

ID	Lat	Long	Nb	*NAe*	*v*	*End*	*S´*
M10x3	-2.65	16.45	0	NA	NA	NA	NA
M10x6	1.85	16.45	0	NA	NA	NA	NA
M5x5	0.35	8.95	0	NA	NA	NA	NA
M5x7	3.35	8.95	3	-0.728	-0.765	-0.722	0.040
M5x8	4.85	8.95	6	-0.397	0.746	-0.722	0.127
M5x9	6.35	8.95	0	NA	NA	NA	NA
M6x3	-2.65	10.45	0	NA	NA	NA	NA
M6x4	-1.15	10.45	2	NA	NA	NA	NA
M6x5	0.35	10.45	12	0.502	1.681	-0.259	0.071
M6x6	1.85	10.45	3	0.738	0.142	1.130	0.345
M6x7	3.35	10.45	23	2.337	0.613	1.694	0.543
M6x8	4.85	10.45	0	NA	NA	NA	NA
M7x3	-2.65	11.95	8	-0.728	-0.765	-0.722	-0.017
M7x4	-1.15	11.95	8	1.187	1.870	1.362	-0.096
M7x5	0.35	11.95	11	-0.728	-0.765	-0.722	-0.127
M7x6	1.85	11.95	6	-0.001	-0.463	1.130	0.016
M8x1	-5.65	13.45	0	NA	NA	NA	NA
M8x4	-1.15	13.45	0	NA	NA	NA	NA
M8x5	0.35	13.45	4	-0.728	-0.765	-0.722	-0.250
M8x7	3.35	13.45	18	-0.728	-0.765	-0.722	-0.289
M9x2	-4.15	14.95	0	NA	NA	NA	NA
M9x5	0.35	14.95	2	NA	NA	NA	NA
M9x7	3.35	14.95	3	-0.728	-0.765	-0.722	-0.362

Megaphrynium macrostachyum

ID	Lat	Long	Nb	*NAe*	*v*	*End*	*S´*
M10x3	-2.65	16.45	30	-0.382	-0.656	-0.050	-0.374
M10x6	1.85	16.45	5	-0.245	0.930	-0.599	0.002
M5x5	0.35	8.95	6	-0.352	0.335	-0.599	0.296
M5x7	3.35	8.95	5	-0.788	-1.016	-0.599	0.388
M5x8	4.85	8.95	14	-0.428	-0.532	-0.011	0.518
M5x9	6.35	8.95	3	-0.788	-1.016	-0.599	0.312
M6x3	-2.65	10.45	3	-0.015	-0.475	0.773	-0.397
M6x4	-1.15	10.45	3	-0.788	-1.016	-0.599	-0.534
M6x5	0.35	10.45	18	2.619	2.017	0.544	0.027
M6x6	1.85	10.45	0	NA	NA	NA	NA
M6x7	3.35	10.45	12	0.333	0.335	0.087	0.609
M6x8	4.85	10.45	2	NA	NA	NA	NA
M7x3	-2.65	11.95	1	NA	NA	NA	NA
M7x4	-1.15	11.95	7	-0.427	-0.718	2.928	-0.033
M7x5	0.35	11.95	16	2.140	0.951	1.715	0.019

M7x6	1.85	11.95	7	0.891	1.764	-0.599	0.366
M8x1	-5.65	13.45	5	-0.788	-1.016	-0.599	-0.487
M8x4	-1.15	13.45	0	NA	NA	NA	NA
M8x5	0.35	13.45	4	-0.098	-0.560	-0.599	-0.549
M8x7	3.35	13.45	15	-0.641	-0.259	-0.599	-0.246
M9x2	-4.15	14.95	0	NA	NA	NA	NA
M9x5	0.35	14.95	1	NA	NA	NA	NA
M9x7	3.35	14.95	5	-0.245	0.930	-0.599	0.083

				Megaphrynium trichogynum			
**ID**	**Lat**	**Long**	**Nb**	***NAe***	***v***	***End***	***S´***
M10x3	-2.65	16.45	4	0.237	0.084	-0.552	0.176
M10x6	1.85	16.45	0	NA	NA	NA	NA
M5x5	0.35	8.95	2	NA	NA	NA	NA
M5x7	3.35	8.95	1	NA	NA	NA	NA
M5x8	4.85	8.95	5	-1.131	-1.047	-0.552	-0.603
M5x9	6.35	8.95	0	NA	NA	NA	NA
M6x3	-2.65	10.45	7	0.458	0.184	-0.552	-0.173
M6x4	-1.15	10.45	10	0.476	0.219	-0.552	-0.148
M6x5	0.35	10.45	27	1.602	2.608	1.485	1.338
M6x6	1.85	10.45	0	NA	NA	NA	NA
M6x7	3.35	10.45	12	-1.131	-1.047	-0.552	-0.603
M6x8	4.85	10.45	0	NA	NA	NA	NA
M7x3	-2.65	11.95	8	0.218	0.084	-0.552	-0.232
M7x4	-1.15	11.95	8	-0.523	-0.387	-0.552	-0.427
M7x5	0.35	11.95	8	-0.523	-0.387	-0.552	1.038
M7x6	1.85	11.95	12	1.886	0.921	1.740	0.479
M8x1	-5.65	13.45	1	NA	NA	NA	NA
M8x4	-1.15	13.45	0	NA	NA	NA	NA
M8x5	0.35	13.45	12	-0.755	-0.586	1.740	-0.341
M8x7	3.35	13.45	14	-0.816	-0.647	-0.552	-0.505
M9x2	-4.15	14.95	0	NA	NA	NA	NA
M9x5	0.35	14.95	0	NA	NA	NA	NA
M9x7	3.35	14.95	0	NA	NA	NA	NA

Supplementary Table 8. Peason correlation of mean endemism (haplotype range < 200 km) between species for grid cell size of 0.75 (upper diagonal) and 1.5° (lower diagonal). (*), marginally significant (p < 0.5); *, significant (p < 0.1); **, highly significant (p < 0.01). NA, no data due to less than 3 individuals per species and grid cell. For abbreviations of species names see Table 1.

	HaloAzu	HauDanck	HauLieb	MarCong	MarIncert	MarMono	MegaMacro	MegaTrich
HaloAzu		NA	NA	0.970**	0.561	-0.256	-0.253	-0.177
HauDanck	0.920**		0.388	NA	-0.218	0.024	-0.294	-0.003
HauLieb	NA	0.396		NA	0.115	0.903*	-0.156	-0.247
MarCong	0.818**	-0.211	NA		0.469	-0.187	-0.200	-0.238
MarIncert	0.216	-0.273	0.566	0.155		0.821*	0.011	0.309
MarMono	-0.303	-0.059	0.975*	-0.247	0.706		-0.125	0.165
MegaMacro	-0.247	-0.047	-0.003	-0.130	-0.015	0.356		-0.108
MegaTrich	-0.288	-0.170	-0.539	-0.222	0.009	0.019	-0.326	

Supplementary Table 9. Results of Mantel test comparing pairwise standardized distinctiveness among grid cells (S'_{ij}) between species pairs for grid cell size of 1.5°. Number of grid cells shared between species (upper diagonal), correlation coefficient (lower diagonal). Significant values (p < 0.05) are written in bold and indicated by a *. For abbreviations of species names see Table 1.

	HaloAzu	*HauDanck*	*HauLieb*	*MarCong*	*MarIncert*	*MarMono*	*MegaMacro*	*MegaTrich*
HaloAzu		6	5	11	6	7	11	8
HauDanck	0.23		6	9	4	7	8	9
HauLieb	-0.14	-0.36		7	4	4	6	7
MarCong	**0.56***	-0.17	-0.32		7	9	12	11
MarIncert	0.13	0.13	-0.01	-0.38		5	5	6
MarMono	-0.13	0.21	0.53	0.32	-0.41		9	9
MegaMacro	0.07	-0.19	0.04	**0.56***	0.06	**0.48***		9
MegaTrich	0.32	0.08	-0.32	-0.14	0.01	-0.05	-0.04	

1.2. Suplementary Figures

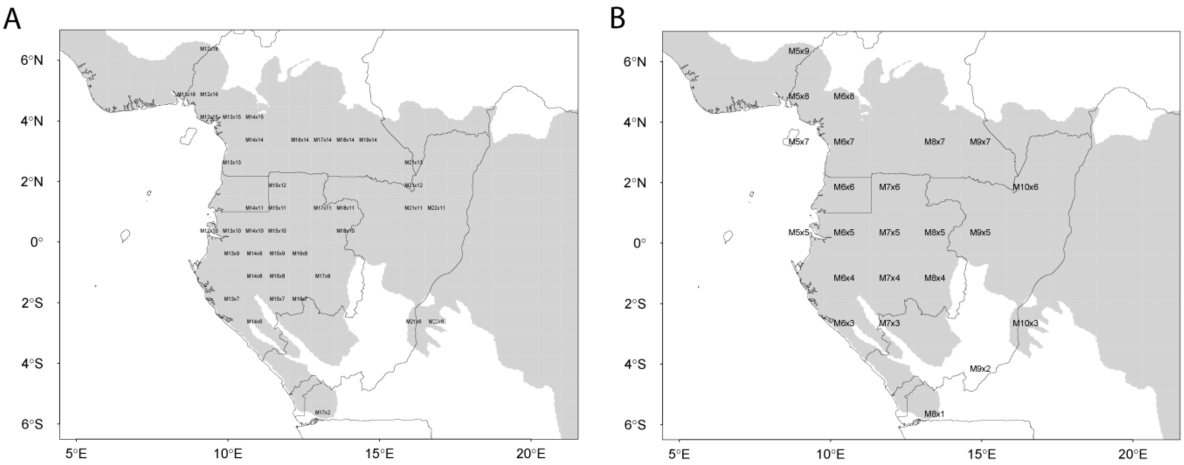

Supplementary Figure 1. Grid systems used to subdivide Lower Guinea. (A) cell sizes: 0.75°-side length, (B) cell sizes: 1.5°-side length.

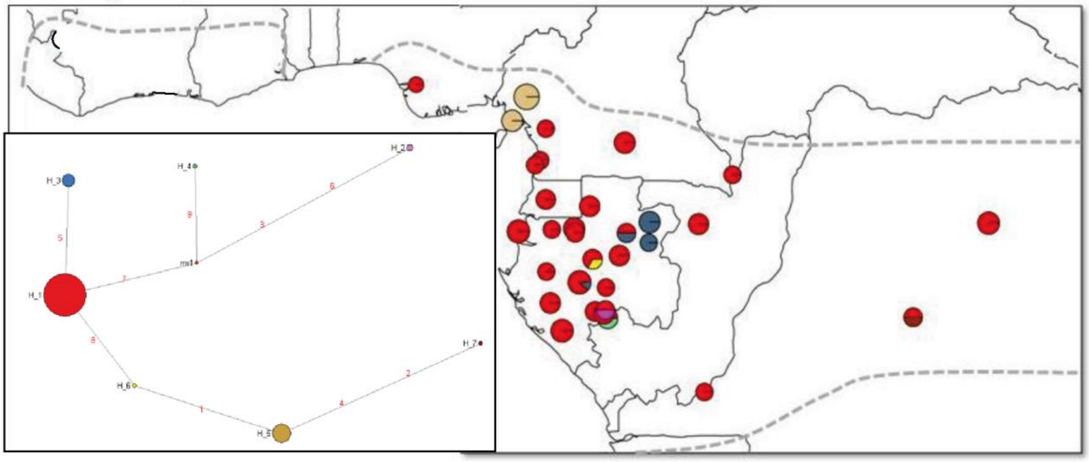

Supplementary Figure 2. Geographic distribution of chloroplast haplotypes and haplotype network based on psbA-trnH2 for *Marantochloa congensis*. grey hatched line: species distribution range.

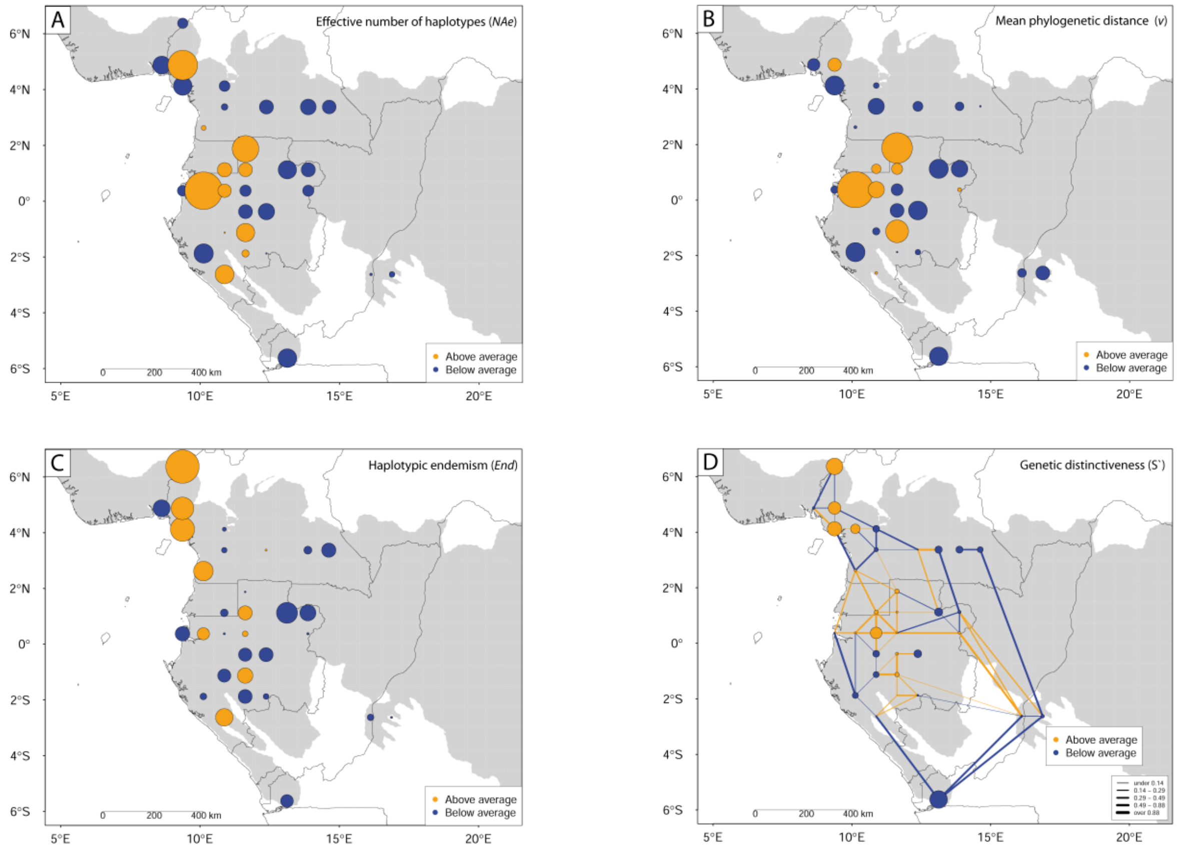

Supplementary Figure 3. Geographic distribution of standardized (i.e. centered and reduced) genetic diversity, endemism and distinction averaged over the eight Marantaceae species in Lower Guinea for grid cell size 0.75°. (A) effective number of haplotypes, (B) mean phylogenetic distance between individuals (v), (C) haplotypic endemism (haplotype range < 200 km), (D) genetic distinctiveness of each grid cell (S´).

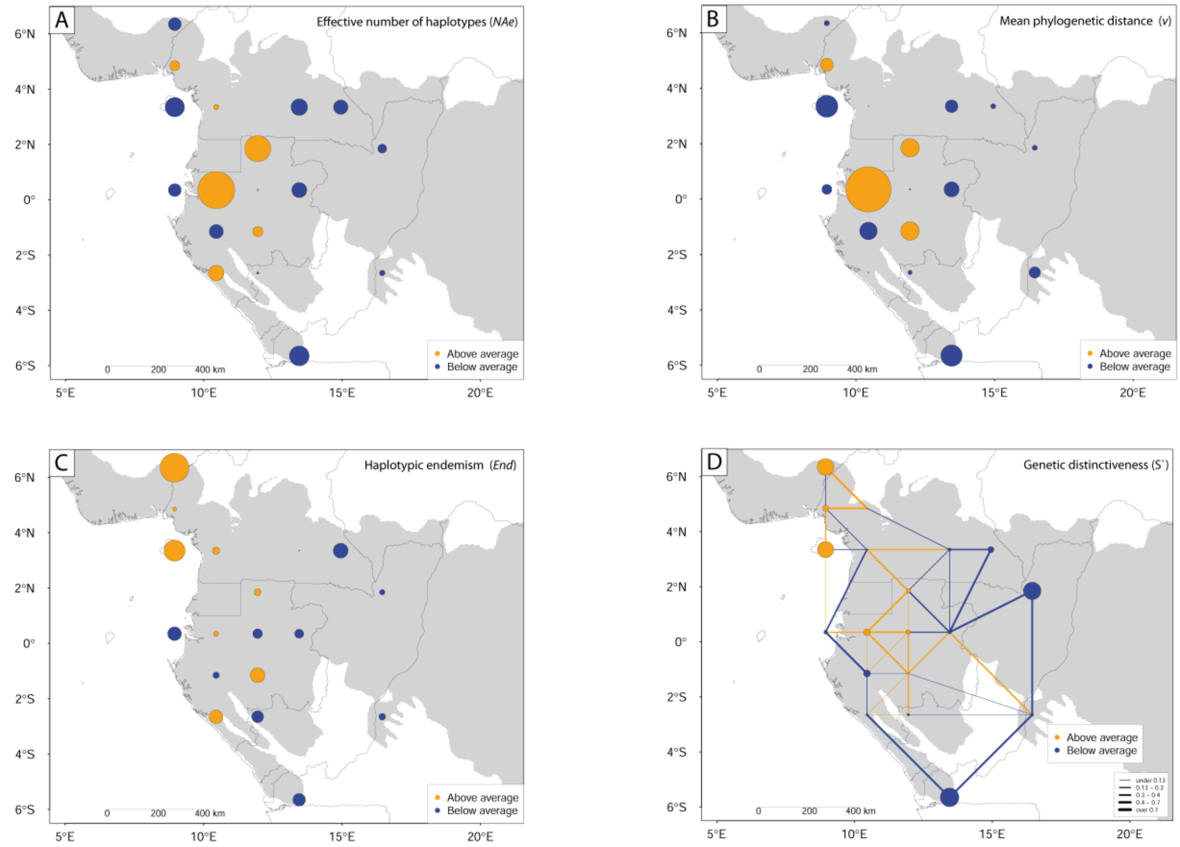

Supplementary Figure 4. Geographic distribution of standardized (i.e. centered and reduced) genetic diversity, endemism and distinction averaged over the eight Marantaceae species in Lower Guinea for grid cell size 1.5°. (A) effective number of haplotypes, (B) mean phylogenetic distance between individuals (v), (C) haplotypic endemism (haplotype range < 200 km), (D) genetic distinctiveness of each grid cell (S´).

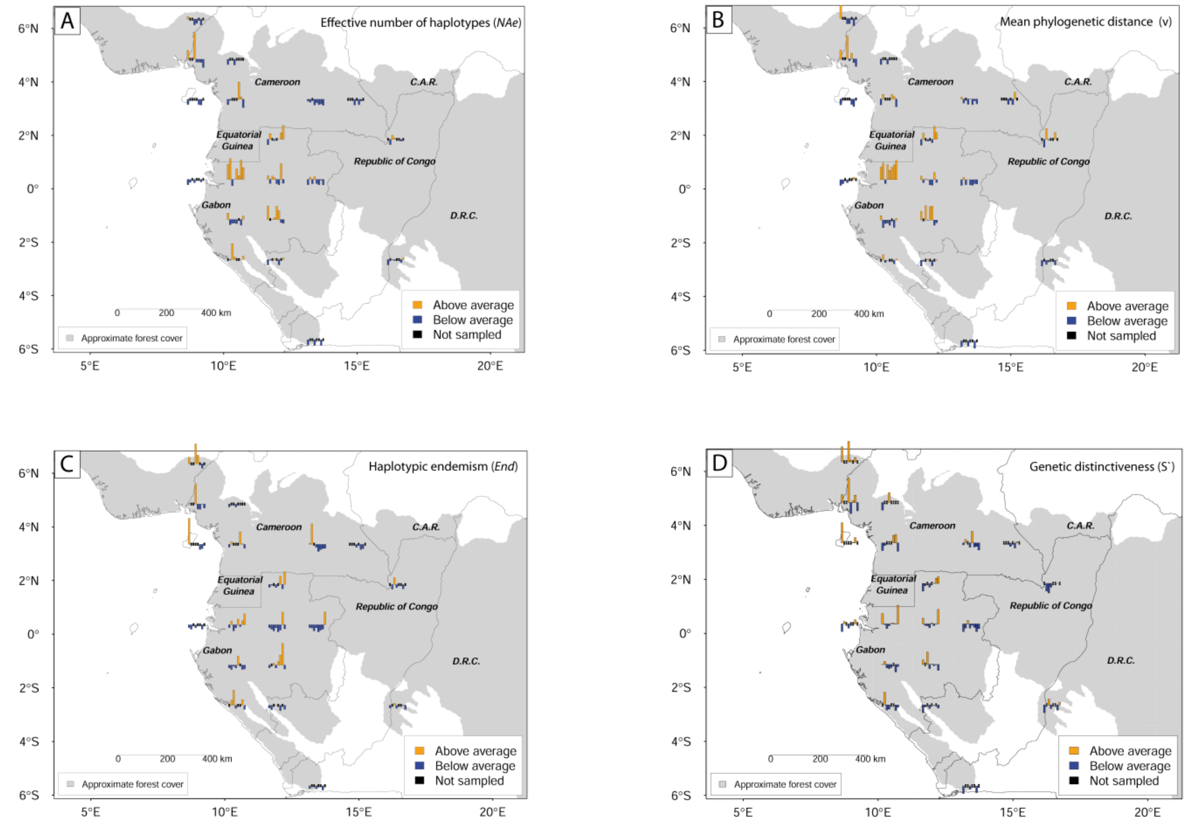

Supplementary Figure 5. Geographic distribution of standardized (i.e. centered and reduced) genetic diversity and endemism for eight Marantaceae species in Lower Guinea for grid cell size 1.5°. (A) effective number of haplotypes (*NAe*), (B) mean phylogenetic distance between individuals (v), (C) haplotypic endemism (*End*, haplotype range < 200 km). (D) Genetic distinctiveness of each grid cell (S´, 1.5°). Distinctiveness above or below average is based on standardized pairwise genetic distance (S'_{kij} computed for each species) among populations where genetic distance is estimated as the number of mutational steps between two individuals drawn from two populations (v_{ij}). Species along barplots from left to right are: *Halopegia azurea*, *Haumania danckelmaniana*, *H. liebrechtsiana*, *Marantochloa congensis*, *M. incertifolia*, *M. monophylla*, *Megaphrynium macrostachyum* and *Mega. trichogynum.*

2. References[1]

[1] Provide the doi when available, and ALL complete author names.
